# Combination Therapy of Phage vB_KpnM_P-KP2 and Gentamicin Combats Acute Pneumonia Caused by K47 Serotype *Klebsiella pneumoniae*

**DOI:** 10.3389/fmicb.2021.674068

**Published:** 2021-04-22

**Authors:** Zijing Wang, Ruopeng Cai, Gang Wang, Zhimin Guo, Xiao Liu, Yuan Guan, Yalu Ji, Hao Zhang, Hengyu Xi, Rihong Zhao, Lanting Bi, Shanshan Liu, Li Yang, Xin Feng, Changjiang Sun, Liancheng Lei, Wenyu Han, Jingmin Gu

**Affiliations:** ^1^Key Laboratory of Zoonosis Research, Ministry of Education, College of Veterinary Medicine, Jilin University, Changchun, China; ^2^College of Animal Science and Technology, Jilin Agricultural University, Changchun, China; ^3^Department of Clinical Laboratory, The First Hospital of Jilin University, Changchun, China; ^4^Department of Chinese Journal of Veterinary Science, Jilin University, Changchun, China; ^5^Jiangsu Co-Innovation Center for the Prevention and Control of Important Animal Infectious Disease and Zoonose, Yangzhou University, Yangzhou, China

**Keywords:** bacteriophage, *Klebsiella pneumoniae*, genome sequencing, bioinformatics analysis, phage therapy

## Abstract

*Klebsiella pneumoniae* (*K. pneumoniae*) is an important nosocomial and community acquired opportunistic pathogen which causes various infections. The emergence of multi-drug resistant (MDR) *K. pneumoniae* and carbapenem-resistant hypervirulent *K. pneumoniae* (CR-hvKP) has brought more severe challenge to the treatment of *K. pneumoniae* infection. In this study, a novel bacteriophage that specifically infects *K. pneumoniae* was isolated and named as vB_KpnM_P-KP2 (abbreviated as P-KP2). The biological characteristics of P-KP2 and the bioinformatics of its genome were analyzed, and then the therapeutic effect of P-KP2 was tested by animal experiments. P-KP2 presents high lysis efficiency *in vitro*. The genome of P-KP2 shows homology with nine phages which belong to “*KP15 virus*” family and its genome comprises 172,138 bp and 264 ORFs. Besides, P-KP2 was comparable to gentamicin in the treatment of lethal pneumonia caused by *K. pneumoniae* W-KP2 (K47 serotype). Furthermore, the combined treatment of P-KP2 and gentamicin completely rescued the infected mice. Therefore, this study not only introduces a new member to the phage therapeutic library, but also serves as a reference for other phage-antibiotic combinations to combat MDR pathogens.

## Introduction

As the second-ranked nosocomial infection-causing-pathogens, *Klebsiella pneumoniae* (*K. pneumoniae*) causes fatal systemic infections (Paczosa and Mecsas, 2016). The presence of at least 79 serotypes greatly increases the complexity of treatment for these bacterial infections (Hsu et al., 2013; Pan et al., 2015). What’s more, the existing antibiotics have failed to cure clinical infections caused by multidrug-resistant (MDR) *K. pneumoniae*, such as extended-spectrum β-lactamase (ESBL)-producing and carbapenemase [such as *K. pneumoniae* carbapenemases (KPC), metallo β-lactamases (MBL), and oxacillinase-48-type carbapenemases (OXA-48)]-producing strains (Navon-Venezia et al., 2017). In addition, the recent emergence of carbapenem-resistant hypervirulent *Klebsiella pneumoniae* (CR-hvKP) has further exacerbated the dilemma of antibiotic treatment, provoking the need for alternative therapies (Gu et al., 2018).

Bacteriophages (phages) are bacterial viruses that specifically recognize, infect, and replicate inside a host bacterium. It has been reported that there are more than 10^31^ phage particles in the biosphere. This massive phage diversity has a marked effect on the environment, ecology, and bacterial evolution (Davies et al., 2016). Owing to their specific bactericidal abilities, phages have been considered as therapeutic agents since the early 1920s. However, the development of this therapy has been hampered by the widespread use of antibiotics (Nobrega et al., 2015). Recently, due to the global emergence of multidrug-resistant bacteria, phage therapy has been experiencing a renaissance for its ability to combat their antibiotic-resistant host specifically. Until now, phage therapy have already been developed in various bacterial species including MDR *K. pneumoniae* and have made some achievements. Studies based on mice as animal models have shown that phages have good therapeutic effects on pneumonia (Anand et al., 2020), liver abscess (Lin et al., 2014), burn infection (Chadha et al., 2017), and bacteremia (Kaabi and Musafer, 2019) caused by *K. pneumoniae*. Not only that, phage therapy has also been applied in clinical practice. Medical workers have confirmed that multiple rounds of phage administration have a significant curative effect on refractory urinary tract infections triggered by MDR *K. pneumoniae* (Bao et al., 2020). Although phage therapy has great potential application prospects, both the phage resistance of bacteria and phage elimination by the immune system are the main challenges (Hodyra-Stefaniak et al., 2015; Hampton et al., 2020). However, the combination therapy of phage and antibiotics can effectively increase the sensitivity of target strains to antibiotics and reduce the probability of phage resistance mutations, thereby providing a development direction for controlling bacterial infections caused by MDR strains (Abedon, 2019).

In the present work, a novel myophage that specifically infects *K. pneumoniae* was isolated and named as vB_KpnM_P-KP2 (abbreviated as P-KP2). The biological characteristics of this phage such as morphology and one-step growth curve were measured. Besides, the genetic background of P-KP2 was revealed by bioinformatics analysis, and the powerful therapeutic effect of the phage combined with gentamicin on lung infection caused by *K. pneumoniae* W-KP2 (K47 serotype MDR *K. pneumoniae* strain) was also confirmed.

## Materials and Methods

### Animals

Female C57BL/6J mice (18–20 g) were purchased from Liaoning Changsheng Biotechnology Co., Ltd. (Benxi, Liaoning, China). All animal experiments were performed in strict accordance with the Regulations for the Administration of Affairs Concerning Experimental Animals approved by the State Council of the People’s Republic of China and the Animal Welfare and Research Ethics Committee at University.

### Bacterial Strains and Culture Conditions

*K. pneumoniae* W-KP2 was isolated from sputum provided by Zhimin Guo and confirmed to be *K. pneumoniae* using 16S rRNA sequence analysis after PCR amplification with the universal primers (27F: AGAGTTTGATCCTGGCTCAG and 1429R: GGTTACCTTGTTACGACTT) (Sunagawa et al., 2009). The serotype of *K. pneumoniae* was identified by the universal primer (F: GGGTTTTTATCGGGTTGTAC and R: 5′-3′ TTCAGCTGGATTTGGTGG) according to the previous description (Pan et al., 2013). The minimum inhibitory concentration (MIC) of antibiotics listed in Supplementary Table 1 for *K. pneumoniae* W-KP2 (0.5–0.63 MacFarland) was assessed using the VITEK^®^ 2 Compact system with VITEK^®^ 2 AST-GN 09 Card (bioMérieux, Marcy-l’Étoile, France) according to the manufacturer’s instructions. The strain was routinely inoculated in lysogeny broth (LB) broth and then propagated on an orbital shaker (180 rpm/min).

### Phage Isolation, Purification, and Host Spectrum Determination

Phage P-KP2 was isolated by using *K. pneumoniae* W-KP2 as the host strain from sewage samples collected from the Changchun sewer system (43°92′ N, 125°25′ E) (Changchun, Jilin Province, China) according to the previously described method (Ji et al., 2019). In brief, W-KP2 was incubated with sewage samples in LB broth at 37°C overnight. After 15 min of centrifugation (4°C, 10,000 × g) to remove the precipitate, the supernatant was filtered by 0.22-μm filters (Millipore, Billerica, MA, United States). Phage P-KP2 was purified by the double-layer agar plate method and then stored at 4°C or −80°C in glycerol (3:1 [*v/v*]). To determine its host spectrum, 5 μL of phage P-KP2 suspension was spot tested against 80 clinical *K. pneumoniae* strains preserved in the laboratory, by the double-layer agar plate method (Cai et al., 2019b).

### Growth Characteristics of the Phage

To determine the titers of phage P-KP2 corresponding to different multiplicity of infection (MOI), the phage was added to fresh *K. pneumoniae* W-KP2 culture with final concentration of 2.0 × 10^7^ CFU/mL (OD_600_ ≈ 0.4) at different MOIs (phage/bacteria = 0.00000001, 0.0000001, 0.000001, 0.00001, 0.0001, 0.001, 0.01, 0.1, 1, 10, and 100). After the mixed culture was incubated for 5 h at 37°C (180 rpm), the phage titers were measured by the double-layer agar plate method after serial dilution (Gong et al., 2016).

To determine the one-step growth curve of P-KP2, the phage was added to W-KP2 culture (5.0 × 10^5^ CFU/mL) at an MOI of 0.1. After 10 min of incubation at 37°C, the mixture was centrifuged at 12,000 × g for 5 min at 4°C. The pellet was resuspended in 10 mL of fresh LB broth followed by incubation at 37°C with shaking at 180 rpm. Cultures were collected every 10 min for 180 min and were filtered by 0.22-μm filters (Millipore, Billerica, MA, United States). Finally, plaque assays were used to quantify the lysate titers at different time intervals (Gadagkar and Gopinathan, 1980).

### Concentration and Purification of the Phage

Phage P-KP2 was concentrated and purified according to the previous description for morphological observation and genome extraction (Sambrook and Russell, 2006). Briefly, 100 μL of phage P-KP2 suspension (2.0 × 10^7^ PFU/mL) was added to 1 L of *K. pneumoniae* W-KP2 (OD_600_ ≈ 0.4). After shaking culture (180 rpm) for 4 h, the lysates were centrifuged for 20 min (4°C, 4,000 × g) to recover the supernatant, and DNase I and RNase A (1 μg/mL) were added. Followed by incubation at 25°C for 30 min, 1 M NaCl was then added to the supernatants (ice bath for 1 h). After the addition of polyethylene glycol 8000 (PEG8000) (10% [*w/v*]), the mixture was incubated in an ice bath overnight. Next, the phage particles were collected by centrifugation for 10 min (4°C, 10,000 × g) and resuspended in 2 mL SM buffer. The concentrated phage was purified by cesium chloride (CsCl) density gradient centrifugation (4°C, 35,000 × g, 3 h, CsCl gradient: 1.32, 1.45, 1.50, and 1.70 g/mL), and then the light blue phage band was carefully collected and subjected to dialysis before being stored at 4°C.

### Morphological Observation of the Phage

After negative staining with phosphotungstic acid (PTA) (2% [*w/v*]), the morphology of the concentrated phage (2.5 × 10^10^ PFU/mL) was examined using a transmission electron microscope (TEM) (JEOL JEM-1200EXII, Japan Electronics and Optics Laboratory, Tokyo, Japan) at an acceleration voltage of 80 kV.

### Sequencing and Bioinformatics Analysis of the Phage Genome

The genome was extracted from the concentrated and purified phage preparations using a viral genome extraction kit (Omega Bio-Tek Inc., Doraville, GA, United States) [Quality criteria for DNA samples: OD_260__/__280_ = 1.8–2.0, OD_260__/__230_ = 2.0–2.2, RIN = 6.0–8.0, ≥10 μg in total (≥100 ng/μL)]. Whole-genome sequencing was performed by using Illumina HiSeq 2500 sequencing. The genome sequences were assembled using Roche Newbler v.2.8 (Margulies et al., 2005). Potential open reading frames (ORFs) were identified using GeneMarkS (Georgia Institute of Technology, Atlanta, GA, United States) [parameter settings: (sequence type: phage; output format for gene prediction: LST; output options: gene nucleotide sequence)] (Besemer et al., 2001). The gene alignment and ORF annotation were performed using BLASTN (nucleotide collection database, Megablast) and BLASTP [non-redundant protein sequence database, PSI-BLAST (Threshold = 0.005)], respectively, from the National Center for Biotechnology Information (NCBI) (Altschul et al., 1997). The schematic of the phage genome with predicted ORFs was generated using CLC Main Workbench, version 8.0.1 (CLC Bio-Qiagen, Aarhus, Denmark). The circular view of the phage genome was illustrated using CGView^1^ (Stothard and Wishart, 2005) and termini of the phage was identified by PhageTerm^2^ (Garneau et al., 2017). Genome comparisons among P-KP2 and homologous phages were performed by Mauve 2.3.1 (Darling et al., 2004). After genes of terminase large subunit, major capsid protein and DNA polymerase were aligned by ClustalW, phylogenetic trees of phages were constructed using Neighbor-Joining Method (100 bootstrap replicates) by PHYLIP (version 3.697) (Shimada and Nishida, 2017). Additionally, domain analyses were performed by HHpred at MPI bioinformatics Toolkit^3^ (Zimmermann et al., 2018).

### Therapeutic Effects of P-KP2 and Gentamicin Against *K. pneumoniae* W-KP2

After *K. pneumoniae* W-KP2 was cultured to exponential growth phase (2.0 × 10^9^ CFU/mL), the bacterial solution was concentrated by centrifugation and suspended in phosphate buffered saline (PBS) (adjust to 5.0 × 10^10^ CFU/mL). To determine the virulence of W-KP2, mice were anesthetized intraperitoneally with ketamine (100 mg/kg) and xylazine (10 mg/kg) followed by intranasal inoculation with 50 μL of W-KP2 suspension of different dilutions (5.0 × 10^5^, 5.0 × 10^6^, 5.0 × 10^7^, 5.0 × 10^8^, 1.0 × 10^9^, or 2.5 × 10^9^ CFU/mouse, *n* = 10 per group), and then the minimum bacterial dose that triggered 100% death within 7-days [minimum lethal dose (MLD)] was determined (Xia et al., 2016). Finally, all surviving mice were euthanized by intravenous injection of Fatal Plus (sodium pentobarbital) (100 mg/kg).

The therapeutic effects of phage P-KP2, gentamicin and phage-antibiotic combination in the treatment of acute pneumonia caused by *K. pneumoniae* W-KP2 were evaluated based on the mouse model. Mice were challenged intranasally with 2 × MLD (1.0 × 10^9^ CFU/mouse) of W-KP2 in reference to our previous study (Cheng et al., 2017). At 1 h post infection, mice were treated intranasally with P-KP2 after concentration (1.0 × 10^7^ PFU/mouse, 1.0 × 10^8^ PFU/mouse or 1.0 × 10^9^ PFU/mouse), gentamicin (1.5 mg/kg), or P-KP2 (1.0 × 10^9^ PFU/mouse) in combination with gentamicin (gentamicin was administered intranasally at 30 min after P-KP2 administration), respectively. The untreated group was administered with PBS buffer after W-KP2 challenge. The survival rate, body weight and health status in each group were recorded within 7 days follow-up period.

Three mice selected randomly from each treatment group (*n* = 30 per group) at every time point were euthanized using Fatal Plus (sodium pentobarbital) (100 mg/kg) every 24 h (lasting for 7 days) after infection. Blood was collected from the tail vein of these mice and then the bacterial load was determined by plating after serial dilution. Their lungs were carefully removed and photographed. After fixation with 4% formalin, the left lungs were embedded with paraffin and stained with hematoxylin and eosin (H&E) (Feldman and Wolfe, 2014), followed by histopathological analysis through a microscope (Olympus CX-41; Olympus America, Center Valley, PA, United States). After weighing, the right lung tissues were immediately lysed and homogenized, then the lung homogenates were serially diluted and bacterial loads were determined by plating. Simultaneously, the phage titers in lung tissues were detected using the double-layer agar plate method. For enzyme-linked immunosorbent assay, the supernatants of lung homogenates were obtained according to our previous method (4°C, 5,000 × g, 10 min) (Cai et al., 2019a). The concentrations of interleukin 1β (IL-1β), interleukin 6 (IL-6), tumor necrosis factor-α (TNF-α), and interferon-γ (IFN-γ) in the supernatants were measured by ELISA kits (eBioscience, San Diego, CA, United States) according to the manufacturer’s instructions.

### Statistical Analysis

Survival curve analyses were performed using log-rank (Mantel–Cox) test. While other statistical data presented in this study were processed by One-way analysis of variance (ANOVA). GraphPad Prism 6 (GraphPad Software, Inc., San Diego, CA, United States) was utilized for chart generation. *P* < 0.05 were considered statistically significant. Error bars represented standard error of the mean.

## Results

### The Characteristics of *K. pneumoniae* W-KP2 and Phage P-KP2

Since sequencing result of W-KP2 *wzc* gene was consistent with K47 serotype reference sequences, *K. pneumoniae* W-KP2 was identified as belonging to the K47 serotype (Supplementary Figure 1), which was resistant to the antibiotics listed in Supplementary Table 1, except for gentamicin, indicating that it is a MDR strain that may lead to ineffective antibiotic treatment.

**FIGURE 1:**
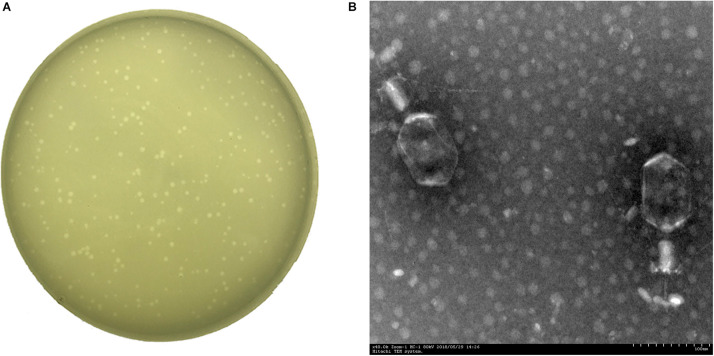
The morphology of phage P-KP2. **(A)** The plaques formed by phage P-KP2 on the lawns of *K. pneumoniae* W-KP2. **(B)** The morphology of phage P-KP2 was determined by transmission electron microscopy (TEM). The scale bars represent 100 nm.

A *Klebsiella* phage named vB_KpnM_P-KP2 (abbreviated as P-KP2) was isolated using *K. pneumoniae* W-KP2 in this study, and the initial titer of phage P-KP2 obtained after phage enrichment was 2.0 × 10^7^ PFU/mL. The phage formed small circular translucent plaques (diameter < 1 mm) on the lawns of *K. pneumoniae* W-KP2 (Figure 1A). P-KP2 showed a prolate head with icosahedral structure (105 ± 5 nm in length and 79 ± 3 nm in diameter) and a long contractile tail (100 ± 10 nm) with a baseplate as observed with TEM (Figure 1B), which indicated that it belongs to the family *Myoviridae*. Among the 80 *K. pneumoniae* strains tested, phage P-KP2 could form both spots and plaques on 10 of them, including W-KP2 (Supplementary Table 2).

**FIGURE 2:**
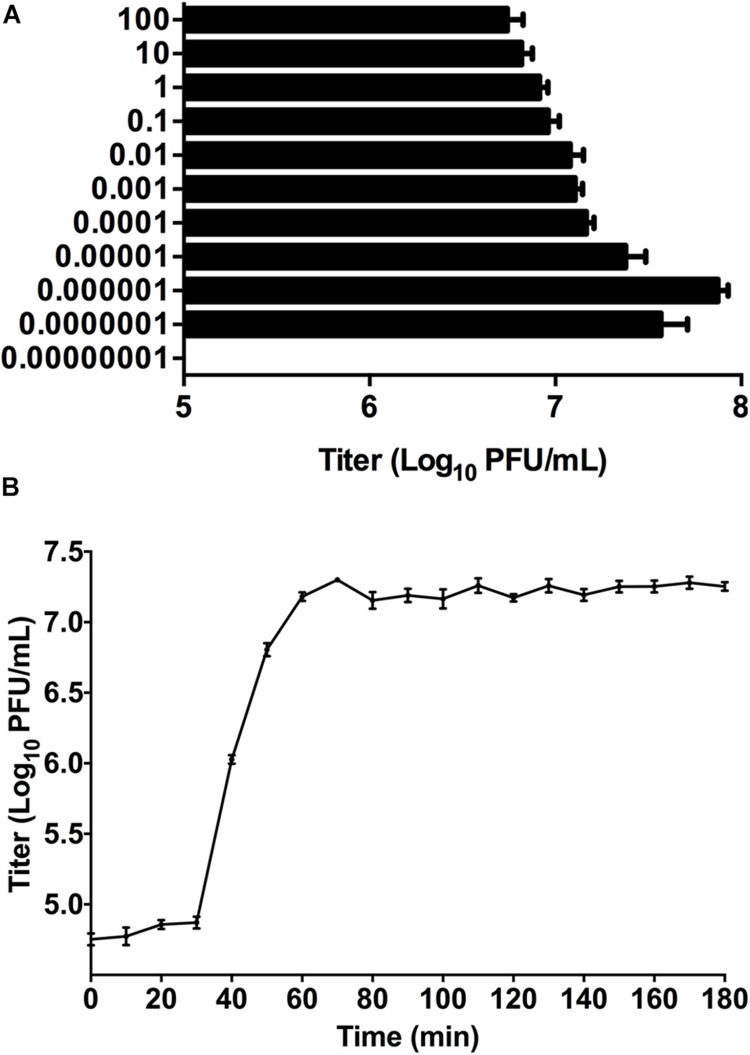
The growth characteristics of P-KP2. **(A)** Titers of the phage under different MOI (phage/bacteria = 0.00000001, 0.0000001, 0.000001, 0.00001, 0.0001, 0.001, 0.01, 0.1, 1, 10, and 100), as indicated in the Y-axis. At the MOI of 0.000001, P-KP2 reached maximum titers. **(B)** One-step growth curve of P-KP2 was carried out at MOI = 0.1. Each data is shown as means ± SEM from three biological experiments.

Phage titer of P-KP2 after propagation was highest at MOI 0.000001 with a titer of 7.5 × 10^7^ PFU/mL (Figure 2A). Besides, the latent period of this phage was 30 min, after which the number of viral particles was rapidly increased. The proliferation of P-KP2 took about another 30 min to reach the growth plateau phase with a burst size of 204 PFU/cell (Figure 2B).

### Bioinformatics Analyses of P-KP2 Genome

**FIGURE 3:**
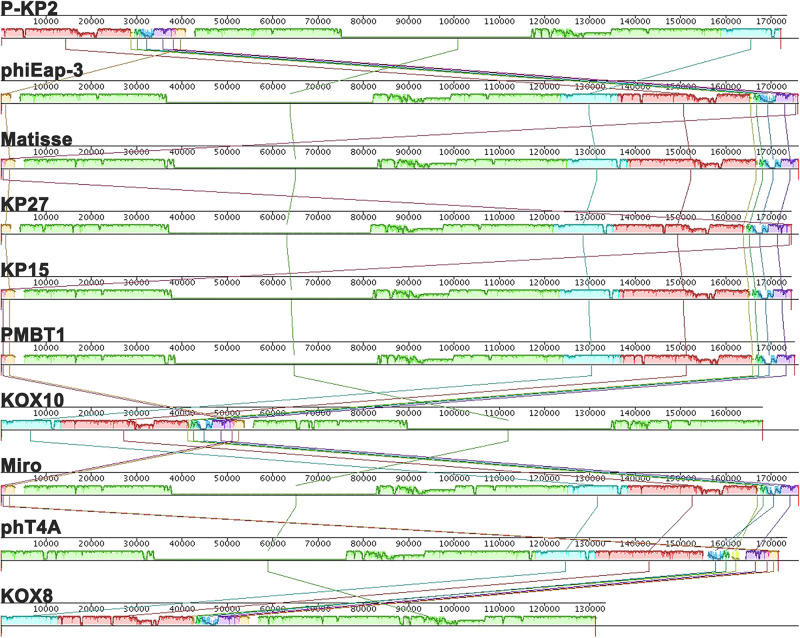
Multiple genome alignments among P-KP2 and homologous phages. The graph was generated by Mauve software. To present the average conservation level in the genome sequence, the similarity of regions is indicated by the height of the bars. Fragment that are not aligned or specific to a particular genome are represented by white areas. Regions of homologous DNA shared by two or more genomes are defined as a local collinear blocks (LCBs), represented by boxes with the identical colors.

**FIGURE 4:**
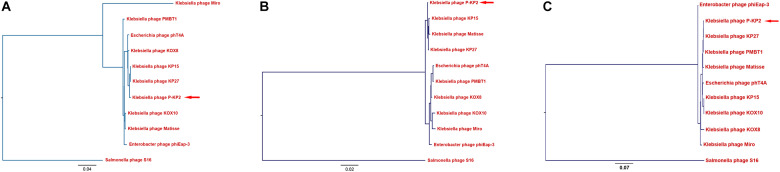
Phylogenetic analyses of P-KP2 and homologous phages. **(A)** Phylogenetic tree based on terminase large subunit genes. **(B)** Phylogenetic tree based on genes of major capsid protein. **(C)** Phylogenetic tree based on genes of DNA polymerase. These sequences were compared using ClustalW, and the phylogenetic tree was generated by PHYLIP version 3.697 (Neighbor-Joining Method, 100 bootstrap replicates). Salmonella phage S16 was selected as the outgroup.

The genome of phage P-KP2 is a linear double-stranded DNA that is comprised of 172,138 bp with a G + C content of 41.9%, and the termini of the phage genome is located at 38,590 bp (Supplementary Figure 2). Comparative analysis of the complete nucleotide sequence indicates that several phages show high similarity to P-KP2, including *Enterobacter* phage phiEap-3 (Zhao et al., 2019), *Klebsiella* phage Matisse (Provasek et al., 2015), *Klebsiella* phage KP27, *Klebsiella* phage KP15, *Klebsiella* phage PMBT1 (Koberg et al., 2017), *Klebsiella* phage KOX10, *Klebsiella* phage Miro (Mijalis et al., 2015), *Escherichia* phage phT4A (Pereira et al., 2017), and *Klebsiella* phage KOX8 (Table 1), which can be classified as a member of pseudo-T-even myophages which belong to “*KP15likevirus*” genus. Multiple genome alignment confirmed that these phages have parallel gene functional modules (Figure 3). Phylogenetic analyses of the large terminase subunits, major capsid proteins and DNA polymerases showed that P-KP2 has close evolutionary relationships with *Klebsiella* phage KP27 (Figure 4).

**TABLE 1:** Global genome comparison of P-KP2 with homologous phages.

	P-KP2	phiEap-3	Matisse	KP27	KP15
Host strain type	*Klebsiella pneumoniae*	*Enterobacter aerogenes*	*Klebsiella pneumoniae*	*Klebsiella pneumoniae*	*Klebsiella pneumoniae*
GenBank number	MT157285.1	KT321315.1	KT001918.1	HQ918180.1	GU295964.1
G + C content	41.9%	42.0%	41.8%	41.8%	41.8%
Genome size (bp)	172,138	175,814	176,081	174,413	174,436
Identity of P-KP2 BLASTN	100%	98%	99%	99%	99%
Query coverage of P-KP2	100%	97%	97%	94%	96%

	**PMBT1**	**KOX10**	**Miro**	**phT4A**	**KOX8**

Host strain type	*Klebsiella pneumoniae*	*Klebsiella oxytoca*	*Klebsiella pneumoniae*	*Escherichia coli*	*Klebsiella oxytoca*; *Klebsiella pneumoniae*
GenBank number	NC_042138.1	MN101223.1	KT001919.1	KX130727.1	MN101221.1
G + C content	41.9%	41.7%	41.8%	41.7%	41.9%
Genome size (bp)	175,206	168,074	176,055	170,698	131,200
Identity of P-KP2 BLASTN	99%	98%	97%	99%	99%
Query coverage of P-KP2	98%	91%	96%	90%	73%

**FIGURE 5:**
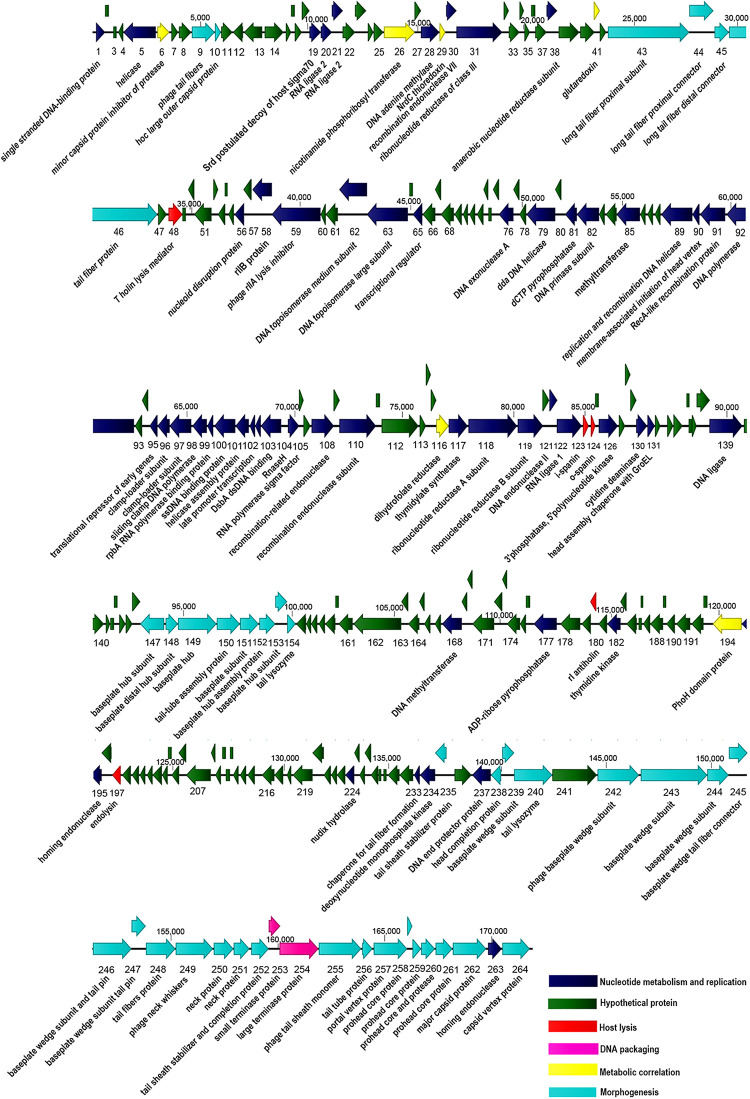
Graphical representation of the phage P-KP2 genome. 264 ORFs and the direction of transcription are presented as arrows. Proposed modules are based on hypothetical functions predicted through bioinformatic analysis. The genome map was generated using CLC Main Workbench (version 8.0.1).

The complete genome of P-KP2 includes 264 predicted ORFs. Among all the ORFs, 120 are transcribed in one orientation, and 144 are transcribed in the opposite orientation; their arrangement at the whole-genome level was mapped (Figure 5). All predicted proteins were examined for similarity to known sequences deposited in the public databases of NCBI, and the obtained information was used to provide detailed annotations of the phage proteome. From Figure 5 and Supplementary Table 3, it can be seen that 106 ORFs share apparent database matches with known functions and the rest 158 ORFs are assigned as hypothetical proteins. Aside lysogeny modules, morphogenesis modules (ORFs 9, 10, 43–46, 147–154, 235, 238–240, 242–252, 255–262, and 264) and DNA packaging modules (ORFs 253–254) can be clearly identified. However, many ORFs are widely distributed in the phage genome without forming an obvious module, ORFs that are associated with nucleotide metabolism and replication and ORFs that are related to metabolic correlation (Figure 5). The host lysis system of P-KP2 is composed of four parts, T holin lysis mediator (ORF48), spanin (ORF123 and ORF124), antiholin (ORF180), and endolysin (ORF197), which is consistent with the characteristics of the lysis modules of “*KP15likevirus*” genus (Zhao et al., 2019).

Most of the gene coding sites of P-KP2 show extremely high homology with their homologous phage, but there are some exceptions. Several loci (including ORFs 3, 20–21, 85, 135–136, and 206 of P-KP2) show different dissimilarity with homologous phages due to the deletion or insertion of a few bases. For example, P-KP2 gp85 (54,624–55,700 bp) has >98% coverage with homologous phages listed in Table 1 at nucleotide level. However, P-KP2 ORF85 shows only 63–72% coverage with these phages. Actually, “C54409” and “T54410” in P-KP2 were inserted compared with homologous phages. Additionally, lacking one “C” between G11040 and C11041 compared with homologous phages, ORF20 and ORF21 were both predicted as parts of RNA ligase 2.

As a putative tail fiber protein, P-KP2 ORF46 shows less similarity with homologous phages either at the nucleotide or at the protein level (Supplementary Figure 3). HHPred analysis shows that ORF46 has some identity with tail spike of Enterobacteria phage Mu (976-1050 residues, PDB ID: 3VTN_A).

### P-KP2 Shows Protective Effects Against *K. pneumoniae* Infection in a Mouse Model

**FIGURE 6:**
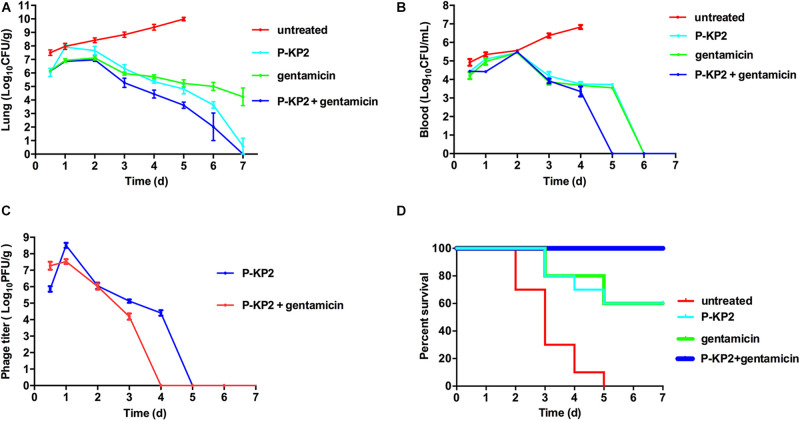
Therapeutic effects of P-KP2 and gentamicin on *K. pneumoniae in vivo*. All the mice were challenged intranasally with 1.0 × 10^9^ CFU/mouse of *K. pneumoniae* W-KP2. After 1 h post infection, they were intranasally treated with P-KP2 (1.0 × 10^9^ PFU/mouse), gentamicin (1.5 mg/kg), or phage-antibiotic combination (gentamicin was administered intranasally at 30 min after P-KP2 administration), respectively. The untreated group was administered with PBS under the same conditions. **(A)** Bacterial loads in the lungs. At every 24 h (lasting for 7 days) after W-KP2 infection, the right lungs of the euthanized mice in each group were carefully separated, weighed and homogenized. Then the bacterial loads in the lung homogenates were detected after serial dilution (*n* = 3). **(B)** Bacterial loads in blood. Peripheral blood samples (10 μL) were obtained from the caudal veins of the anesthetized mice (*n* = 3). **(C)** Phage titers in the lungs. Phage titers of lung homogenates of each group were detected after serial dilution (*n* = 3). The above data represent the mean ± SEM of triplicate experiments. **(D)** Survival rates. Survival rates of W-KP2-infected mice in each group were determined. Each group contained ten mice. Statistical analysis was performed using the Kaplan-Meier method [*P* < 0.0001, log-rank (Mantel-Cox) test].

**FIGURE 7:**
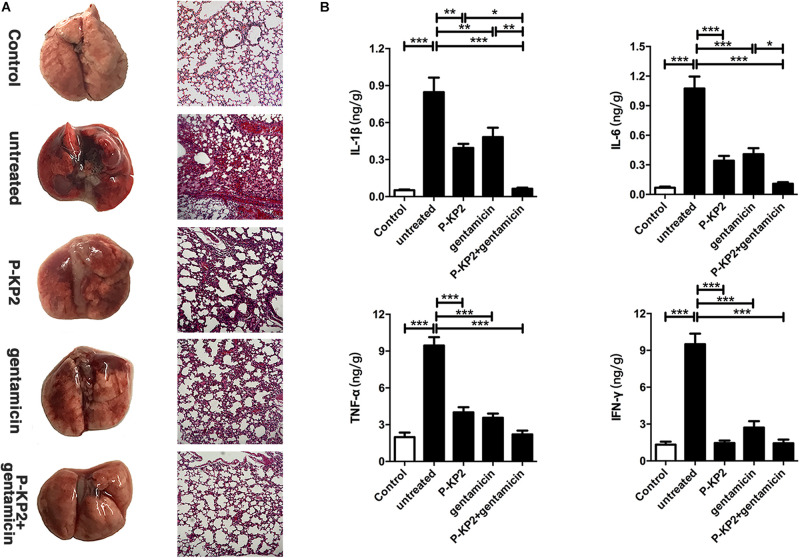
Pathological changes and cytokine levels of mice in different groups. All the mice were challenged intranasally with 1.0 × 10^9^ CFU/mouse of *K. pneumoniae* W-KP2. After 1 h post infection, they were intranasally treated with P-KP2 (1.0 × 10^9^ PFU/mouse), gentamicin (1.5 mg/kg), or phage-antibiotic combination (gentamicin was administered intranasally at 30 min after P-KP2 administration), respectively. The untreated group was administered with PBS under the same conditions. **(A)** Pathological observation. At 72 h after W-KP2 infection, the lungs of the euthanized mice in each group were photographed after careful removal, and the sections of left lung tissues were stained with H&E (magnification, ×100). Lung tissue of healthy mice was served as a control. **(B)** Determination of cytokine levels. At 72 h after W-KP2 infection, cytokine levels (IL-1β, IL-6, TNF-α, and IFN-γ) in lung homogenates of mice treated with phage P-KP2, gentamicin, or phage-antibiotic combination were determined. Lung tissue homogenates of healthy mice were served as controls. *, **, and *** represent significant differences at *P* < 0.05, *P* < 0.01, and *P* < 0.001, respectively. Data represent the mean ± SEM of triplicate experiments.

The MLD of intranasal inoculation of *K. pneumoniae* W-KP2 was determined as 5.0 × 10^8^ CFU/mouse. To monitor the therapeutic effect of phage P-KP2 on acute pneumonia caused by W-KP2, mice were infected with 2 × MLD (1.0 × 10^9^ CFU/mouse). At the initial stage of infection (12 h), only a small amount of W-KP2 was colonized. However, without therapeutic intervention, the bacterial load in the lung raised to nearly 10^10^ CFU/g within 5 days (Figure 6A), and a considerable amount of bacteria entered the peripheral blood (6.7 × 10^6^ CFU/mL) (Figure 6B). These untreated mice developed severe congestion and hemorrhage in the lung tissues as the disease progressed, most of the alveolar structures collapsed and partially disappeared, accompanied by fibrotic lesions (Figure 7A and Supplementary Figure 6). Besides, W-KP2 infection significantly induced the up-regulation of pro-inflammatory cytokines, such as IL-1β, IL-6, TNF-α, and IFN-γ in mice at 72 h after infection (Figure 7B). Accompanied by sustained weight loss and declining health status (Supplementary Figure 4), all the mice of this group died within 5 days.

Phage P-KP2 had a concentration-dependent therapeutic effect on lung infection in mice. Neither 1.0 × 10^7^ PFU/mouse nor 1.0 × 10^8^ PFU/mouse of P-KP2 was able to eliminate bacteria from the lungs within 7 days (Supplementary Figure 5A). The phage titer in the lungs of mice treated with low concentration of phage (1.0 × 10^7^ PFU/mouse) was lower than 10^4^ PFU/g, and phages were completely exhausted within 3 days (Supplementary Figure 5C). Moreover, low concentration of phage (1.0 × 10^7^ PFU/mouse) cannot stop bacterial proliferation and lung damage, leading to weight loss, poor health status and only 20% survival rate (Supplementary Figures 4, 5D). When the therapeutic concentration was 1.0 × 10^9^ PFU/mouse, the bacteria loads in the lungs were effectively reduced (Figure 6A) along with the increase of phage titers (Figure 6C). At the same time, the pneumonia symptoms of mice were significantly alleviated and the survival rate increased to 70% according to the results of 7 days monitoring, which was comparable to the therapeutic effect of gentamicin (Figure 6D). The lungs of gentamicin-treated and phage-treated mice showed mild congestion at 72 h post infection, but the texture remained tough and shiny. The histopathological changes of the two treatment groups had many similarities, both of which showed local capillary dilatation and a small amount of collapse in local alveolar walls, but most of the alveolar structures still in their normal morphology (Figure 7A and Supplementary Figure 6). In spite of P-KP2 (1.0 × 10^9^ PFU/mouse) was less able to eliminate bacteria than gentamicin within 3 days of infection, phage treatment was more effective in eliminating residual bacteria from the lungs at the late stage of infection (Figure 6A). Unfortunately, we may not be able to obtain better phage treatment results because higher P-KP2 concentrations cannot be obtained. However, it is heartening that mice treated with both gentamicin and phage showed only transient respiratory symptoms and completely survived (Figure 6D). When P-KP2 was used in combination with gentamicin, the bacteria in the lungs was almost completely eliminated within 6 days (Figure 6A). Moreover, the lung tissue status of the dual treatment group never showed obvious pathological changes (Figure 7A and Supplementary Figure 6). Additionally, at 72 h after infection, the cytokine levels of the double-treated group were comparable to healthy mice, but both gentamicin-treated and phage-treated mice were slightly higher than that in the dual treatment group (Figure 7B). Therefore, for the lung infection in mice caused by W-KP2, the above results not only showed that P-KP2 had a good substitution effect on gentamicin, but also suggested that the combined effect of phage and gentamicin was far superior to that of a single administration.

## Discussion

According to the production of capsular polysaccharides (CPSs), *K. pneumoniae* can be divided into two major types: classic *K. pneumoniae* (cKP) and hypervirulent *K. pneumoniae* (hvKP). Unlike hvKP strains, which are prevalent in people with normal immunity, cKP strains are mainly found in populations with weakened immunity or immunodeficiency. Derived from the sputum, non-capsulated W-KP2 is a typical cKP strain. However, it is only sensitive to gentamicin in the drug sensitivity test including carbapenems, so it can be defined as a typical MDR *K. pneumoniae*. Although the virulence of W-KP2 could not be compared with the hypervirulent strains with capsules (especially K1 and K2 serotypes), high-dose (1.0 × 10^9^ CFU/mouse) challenge through the respiratory tract still caused mice to die within 5 days. Even after 7 days of infection, residual strains could still be detected in gentamicin-treated mice. Therefore, once such MDR *K. pneumoniae* causes clinical infection, especially the immunodeficiency population as the target, it is likely that the lag of antibiotic treatment (appropriate antibiotics and doses need to be explored) will lead to delayed healing and even poor prognosis. What’s more serious is that while medical workers are still struggling with hvKP and MDR-KP, CR-hvKP have quietly approached as a new threat, and this new type of strains are largely derived from the horizontal transfer of virulence plasmids or drug-resistant plasmids among strains that have existed for a long time in hospitals or communities. As confirmed by recent studies, some carbapenemase-producing strains, especially members of serotype K47, converted to CR-hvKP by obtaining pLVPK-like plasmids (Gu et al., 2018). It can be inferred that W-KP2 also has the possibility to evolve into CR-hvKP. Therefore, this study attempted to control the infection caused by W-KP2 *in vivo* through phage therapy and phage-antibiotic combination therapy and provided a solution to prevent such strains from evolving into CR-hvKP.

P-KP2 has extremely high sequence homology with *Enterobacter* phage phiEap-3 (Zhao et al., 2019) and *Escherichia* phage phT4A (Pereira et al., 2017). They all form small plaques with a diameter ≈ 1 mm, but their host spectrums are quite different. The host spectrums of phiEap-3 and phT4A are much wider than that of P-KP2, but they cannot lyse any *K. pneumoniae*. The host spectrum of phages largely depends on the three-dimensional structure of their receptor-binding proteins (RBPs), especially the C-terminus. With the functions of depolymerase or tail spike protein (TSP), most of RBPs are encoded in the tail fiber protein, and a few are located on the base plate or neck of the phage (Pires et al., 2016). In this study, P-KP2 ORF46, a putative tail fiber protein, had been predicted to be RBP of the phage due to its tail spike-like domain (976–1,050 residues, PDB ID: 3VTN_A), indicating that the protein may mediate specific binding of phage to the host outer membrane proteins (Tu et al., 2017). The C-terminus of ORF46 and its homologous proteins have low sequence conservation (Supplementary Figure 3), suggesting that gp46 may be originated from a later evolutionary stage in the process of horizontal gene exchange (Evseev et al., 2020). Besides, the unique C-terminus may determine the huge host spectrum difference between P-KP2 and its homologous phages. In fact, phage P-KP2 can specifically lyse K47 serotype *K. pneumoniae.* The reason for its narrow host spectrum may be that clinical isolates of this serotype are not common.

The high burst size of P-KP2 (204 PFU/cell) may depend on the multi-gene lysis system composed of T holin lysis mediator (ORF48), spanin (ORF123 and ORF124), antiholin (ORF180), and endolysin (ORF197). Antiholin can control the timing of host lysis by regulating holin, thereby gaining time for the replication and assembly of more progeny virions, resulting in a larger burst size of phages with multi-gene lysis systems (Cahill and Young, 2019). With such a high burst size and no harmful genes in the genome, P-KP2 has the potential to become a therapeutic agent for K47 serotype *K. pneumoniae* infection. In our study, P-KP2 treatment (1.0 × 10^9^ PFU/mouse) alone significantly reduced inflammatory responses and pathological changes in mice with acute pneumonia *in vivo*. In addition, the survival rate of the group was comparable to that of treated with gentamicin, but it was significantly better in eliminating residual bacteria from lung tissue. Some recent studies have shown that the combined administration of phages and antibiotics is more effective than monotherapy in treating bacterial infections (Segall et al., 2019). Based on this, we attempted to treat acute pneumonia caused by W-KP2 with a combination of P-KP2 and gentamicin. Encouragingly, not only did all the mice of the combined therapy group survive, but the pathological changes and inflammatory responses in the lungs were maintained at normal levels, possibly due to the effective inhibition of bacterial proliferation and complete elimination of residual bacteria by phage-antibiotic combination therapy. As an important reason for limiting the therapeutic effect of P-KP2 alone, this phage was easily eliminated by the immune system (Supplementary Figure 5C), and gentamicin seems to play a leading role in our combination therapy. However, phages specifically alter the surface structures of bacteria (polysaccharides, outer membrane proteins, etc.), thereby clearing the barriers for the infiltration of some antibiotics, indicating that phages have the effect of enhancing the sensitivity of bacteria to antibiotics (Segall et al., 2019). Therefore, the synergistic effect of phages on antibiotics is the key to the outstanding therapeutic effect of this combination therapy.

From the main way of treating bacterial infections worldwide, it will take time for us to get rid of dependence on antibiotics completely. However, both the combination of high-dose antibiotics and the treatment with new antibiotics may increase suffering and treatment costs for patients. Therefore, in the context of “weak” antibiotic efficacy, it is more realistic to seek ways to enhance the therapeutic effect of “low-grade” antibiotics. In summary, our study provided a clear case for phage-antibiotic combination against MDR *K. pneumoniae* infections and pointed out the direction to curb the emergence of more CR-hvKP, which has important theoretical significance and practical value.

## Conclusion

The threat of MDR *K. pneumoniae* to public health in humans, especially in immunocompromised populations, and its evolution toward CR-hvKP have led to increased public recognition in the substitution or synergism of phage therapy for antibiotics. In this study, MDR *K. pneumoniae* W-KP2 was used as the host to isolate a novel myophage P-KP2 belonging to “*KP15 virus*” family, and then its biological characteristics and genomics information were analyzed. Due to its high burst size and the absence of harmful genes, P-KP2 was analyzed as a candidate therapeutic agent against acute pneumonia caused by W-KP2. When administered with 1.0 × 10^9^ PFU/mouse, the phage proliferated rapidly and completely eliminated the bacteria in the lungs within 7 days, which exceeded the therapeutic effect of gentamicin. More encouragingly, the combination of P-KP2 and gentamicin not only rescued all infected mice, but also effectively inhibited the development of inflammation. Therefore, as the first case of “*KP15 virus*” family phage to be applied for treatment, the processes of P-KP2 proliferation, promotion of bacterial elimination have been elucidated *in vivo*, which not only filled the gap in the phage library against K47 serotype *K. pneumoniae* infection, but also provided a theoretical basis for the subsequent phage-antibiotic combination therapies and blocking the occurrence of more CR-hvKP.

## Data Availability Statement

The completed genome sequence of Klebsiella phage P-KP2 has been deposited in the GenBank database under accession number MT157285.

## Ethics Statement

The animal study was reviewed and approved by the Care and Use of Laboratory Animals of the Jilin University.

## Author Contributions

GW, ZG, YJ, XL, YG, HZ, HX, RZ, LB, SL, and LY assisted in carrying out the experiment. ZW and RC wrote the manuscript. XF, CS, LL, WH, and JG helped with the design of experimental ideas and the revision of manuscripts. All authors contributed to the article and approved the submitted version.

## Conflict of Interest

The authors declare that the research was conducted in the absence of any commercial or financial relationships that could be construed as a potential conflict of interest.

## Footnotes

1 http://wishart.biology.ualberta.ca/cgview/

2 https://sourceforge.net/projects/phageterm

3 https://toolkit.tuebingen.mpg.de/#/tools/hhpred
