# Supplementary material for: Combination Therapy of Phage vB_KpnM_P-KP2 and Gentamicin Combats Acute Pneumonia Caused by K47 Serotype Klebsiella pneumoniae
Source: Front Microbiol. 2021 Apr 22;12:674068. doi: 10.3389/fmicb.2021.674068 (PMC8100603; doi:10.3389/fmicb.2021.674068)
Supplement: Supplementary file 1 [file Data_Sheet_1.pdf]

Supplementary Material

Supplementary Figures

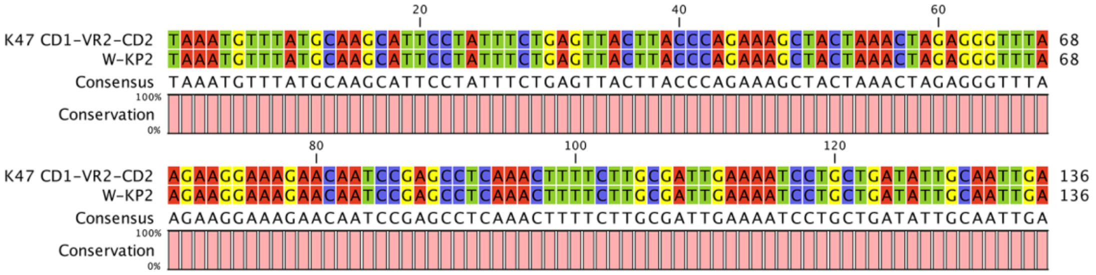

Supplementary Figure 1. Serotype identification of *K. pneumoniae* W-KP2. After alignment with the CD1-VR2-CD2 reference sequence of each serotype *K. pneumoniae* strain, W-KP2 has been identified as a member of K47 serotype strains.

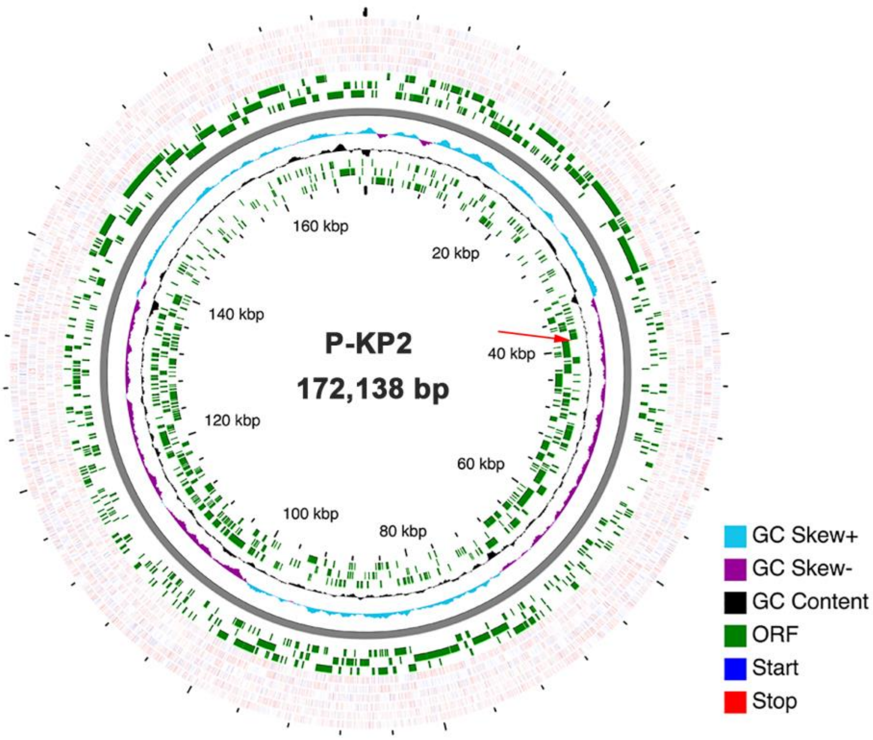

Supplementary Figure 2. Circular view of the P-KP2 genome. The ORFs, G + C content, skew and termini (red arrow) of P-KP2 genome were illustrated. The circle map was generated using CGView (http://wishart.biology.ualberta.ca/cgview/) and the termini of the phage genome was identified as 38,590 bp by PhageTerm (https://sourceforge.net/projects/phageterm).

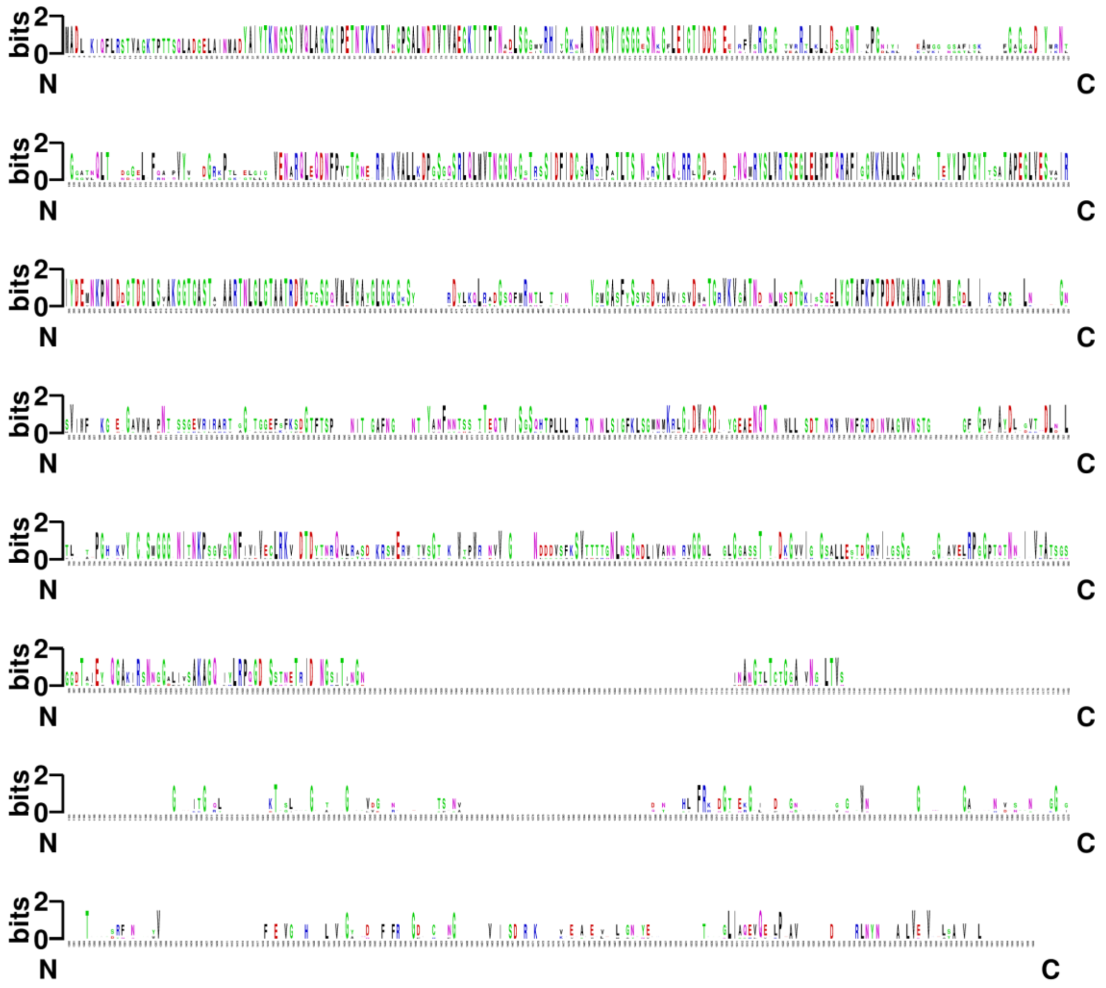

Supplementary Figure 3. Sequence conservation analysis of P-KP2 ORF46 in comparison with other homologous sequences. P-KP2 ORF46, phiEap-3 gp266, KP27 gp286, KP15 gp234 and phT4A gp254 were aligned and displayed as sequence logo, which was created online at http://weblogo.berkeley.edu/logo.cgi.

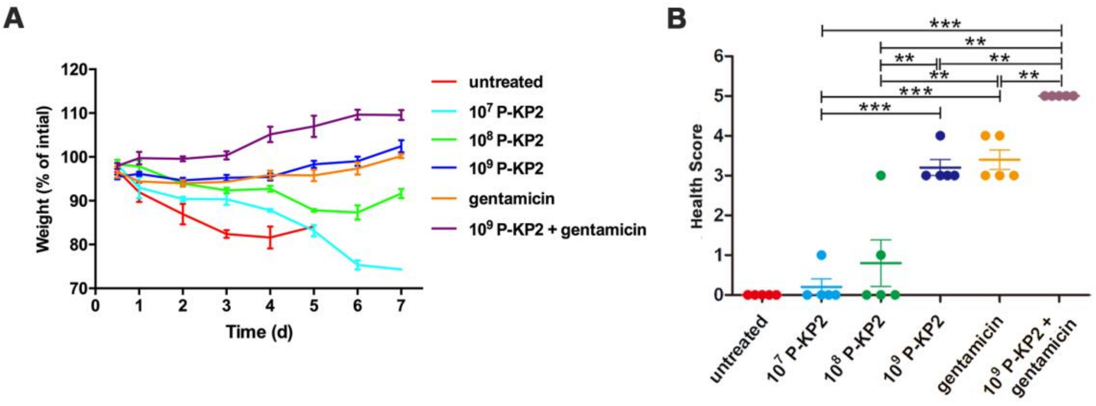

Supplementary Figure 4. Body weight and Health status of mice in different groups. All the mice were challenged intranasally with 1.0 × 10^9 CFU/mouse of *K. pneumoniae* W-KP2. After 1 h post infection, they were treated with different doses of P-KP2 (1.0 × 10^7 PFU/mouse, 1.0 × 10^8 PFU/mouse or 1.0 ×10^9 PFU/mouse), gentamicin (1.5 mg/kg), or phage-antibiotic combination, respectively. The untreated group was administered with PBS under the same conditions. (A) Body weight. Body weight change ([measured weight/initial weight] × 100%) of mice in each group during 7 days was determined (n = 3). (B) Health status. The health status of each group of mice within 7 days was scored on a scale of 5 to 0. Complete health was defined as having almost no symptoms, with a score of 5; mild illness was defined as reduced physical activity and ruffled fur, with a score of 4. Moderate illness was defined as kyphosis and lethargy, with a score of 3. Severe illness was defined as the exudative accumulation around half-closed eyes in addition to the above signs, with a score of 2. The dying state was scored as 1; death was recorded as 0. Each dot represents the health status of a single mouse (n = 5). *, **, and *** represent significant differences at $P < 0.05$, $P < 0.01$, and $P < 0.001$, respectively.

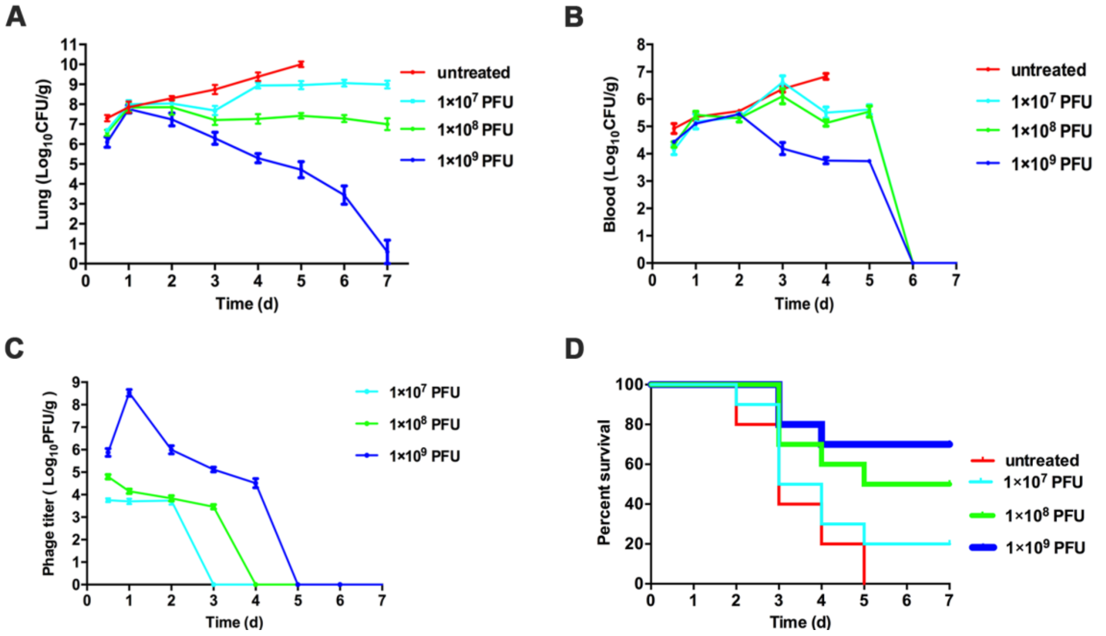

Supplementary Figure 5. Therapeutic effects of W-KP2-infected mice treated with different doses of P-KP2. All the mice were challenged intranasally with 1.0 × 10^9 CFU/mouse of *K. pneumoniae* W-KP2. After 1 h post infection, they were treated with different doses of P-KP2 (1.0 × 10^7 PFU/mouse, 1.0 × 10^8 PFU/mouse or 1.0 ×10^9 PFU/mouse), respectively. The control untreated group was treated administered with PBS under the same conditions. (A) Bacterial loads in the lungs. At every 24 h (lasting for 7 days) after W-KP2 infection, the right lungs of the euthanized mice in each group were carefully separated, weighed and homogenized. Then the bacterial loads in the lung homogenates were detected after serial dilution (n = 3). (B) Bacterial loads in blood. Peripheral blood samples (10 μL) were obtained from the caudal veins of the anesthetized mice (n = 3). (C) Phage titers in the lungs. Phage titers of lung homogenates of each group were detected after serial dilution (n = 3). The above data represent the mean ± SEM of triplicate experiments. (D) Survival rates. Survival rates of mice in each group during 7 days were determined. Each group contained ten mice. Statistical analysis was performed using the Kaplan-Meier method by ($P < 0.0001$, log-rank (Mantel-Cox) test).

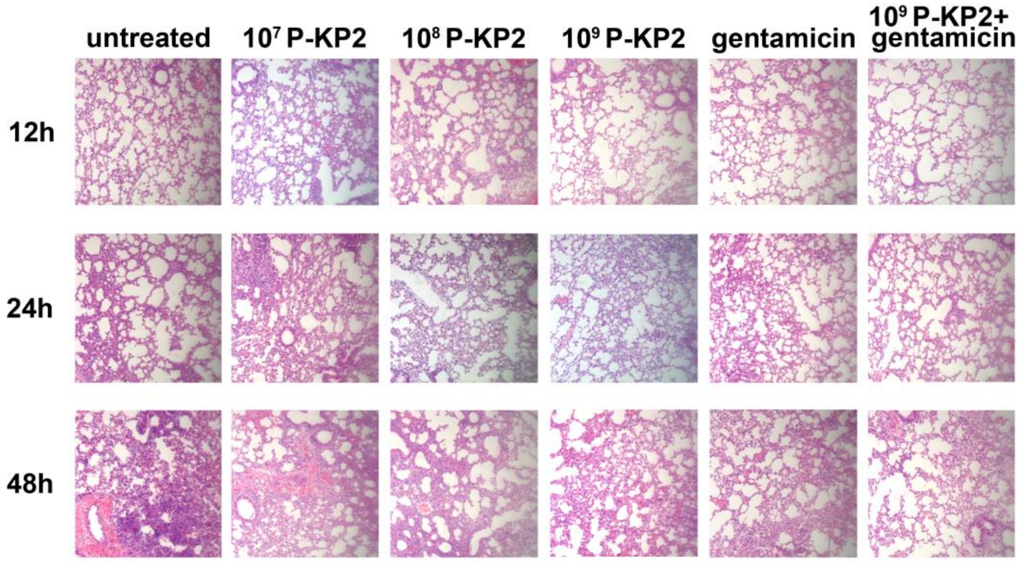

Supplementary Figure 6. Pathological observation at different times after infection. All the mice were challenged intranasally with 1.0 × 10^9 CFU/mouse of *K. pneumoniae* W-KP2. After 1 h post infection, they were treated with different doses of P-KP2 (1.0 × 10^7 PFU/mouse, 1.0 × 10^8 PFU/mouse or 1.0 ×10^9 PFU/mouse), gentamicin (1.5 mg/kg), or phage-antibiotic combination, respectively. The untreated group was administered with PBS under the same conditions. At 12h 24h and 48 h after W-KP2 infection, the lungs of the euthanized mice in each group were photographed after careful removal, and the sections of Left lung tissues were stained with H&E (magnification, ×100).

Supplementary Table S1. The antibiotic-resistance of *K. pneumoniae* W-KP2.

**Antibiotics**	Cefuroxime axetil	Cefepime	Imipenem	Gentamicin	Ampicillin	Cefazolin	Cefuroxime	Piperacillin
**MIC (μg/ml)**	≥64	≥64	≥16	≤1	≥32	≥64	≥64	≥128
**Sensitivity**	R	R	R	S	R	R	R	R

Table S1 (Continued).

**Antibiotics**	Ceftazidime	Ceftriaxone	Meropenem	Ciprofloxacin	Levofloxacin	Cefotetan	Aztreonam	Ampicillin/Sulbactam
**MIC (μg/ml)**	≥64	≥64	≥16	≥4	≥8	≥64	≥64	≥32
**Sensitivity**	R	R	R	R	R	R	R	R

S, Susceptible; R, Resistant.

Supplementary Table S2. The host spectrum of P-KP2

species	ID	spot	plaque
Klebsiella pneumoniae	W-KP2	+	+
Klebsiella pneumoniae	W-KP46	+	+
Klebsiella pneumoniae	W-KP54	+	+
Klebsiella pneumoniae	W-KP60	+	+
Klebsiella pneumoniae	W-KP73	+	+
Klebsiella pneumoniae	W-KP82	+	+
Klebsiella pneumoniae	W-KP83	+	+
Klebsiella pneumoniae	W-KP86	+	+
Klebsiella pneumoniae	W-KP90	+	+
Klebsiella pneumoniae	W-KP93	+	+
Klebsiella pneumoniae	W-KP1	-	-
Klebsiella pneumoniae	W-KP3	-	-
Klebsiella pneumoniae	W-KP4	-	-
Klebsiella pneumoniae	W-KP5	-	-
Klebsiella pneumoniae	W-KP6	-	-
Klebsiella pneumoniae	W-KP7	-	-
Klebsiella pneumoniae	W-KP8	-	-
Klebsiella pneumoniae	W-KP9	-	-
Klebsiella pneumoniae	W-KP10	-	-
Klebsiella pneumoniae	W-KP11	-	-
Klebsiella pneumoniae	W-KP12	-	-
Klebsiella pneumoniae	W-KP13	-	-
Klebsiella pneumoniae	W-KP14	-	-
Klebsiella pneumoniae	W-KP15	-	-
Klebsiella pneumoniae	W-KP16	-	-
Klebsiella pneumoniae	W-KP17	-	-
Klebsiella pneumoniae	W-KP18	-	-
Klebsiella pneumoniae	W-KP19	-	-
Klebsiella pneumoniae	W-KP20	-	-

Klebsiella pneumoniae	W-KP21	-	-
Klebsiella pneumoniae	W-KP22	-	-
Klebsiella pneumoniae	W-KP23	-	-
Klebsiella pneumoniae	W-KP24	-	-
Klebsiella pneumoniae	W-KP25	-	-
Klebsiella pneumoniae	W-KP26	-	-
Klebsiella pneumoniae	W-KP27	-	-
Klebsiella pneumoniae	W-KP28	-	-
Klebsiella pneumoniae	W-KP29	-	-
Klebsiella pneumoniae	W-KP30	-	-
Klebsiella pneumoniae	W-KP31	-	-
Klebsiella pneumoniae	W-KP32	-	-
Klebsiella pneumoniae	W-KP33	-	-
Klebsiella pneumoniae	W-KP34	-	-
Klebsiella pneumoniae	W-KP35	-	-
Klebsiella pneumoniae	W-KP36	-	-
Klebsiella pneumoniae	W-KP37	-	-
Klebsiella pneumoniae	W-KP38	-	-
Klebsiella pneumoniae	W-KP39	-	-
Klebsiella pneumoniae	W-KP40	-	-
Klebsiella pneumoniae	W-KP41	-	-
Klebsiella pneumoniae	W-KP42	-	-
Klebsiella pneumoniae	W-KP43	-	-
Klebsiella pneumoniae	W-KP44	-	-
Klebsiella pneumoniae	W-KP45	-	-
Klebsiella pneumoniae	W-KP47	-	-
Klebsiella pneumoniae	W-KP48	-	-
Klebsiella pneumoniae	W-KP49	-	-
Klebsiella pneumoniae	W-KP50	-	-
Klebsiella pneumoniae	W-KP51	-	-
Klebsiella pneumoniae	W-KP52	-	-
Klebsiella pneumoniae	W-KP53	-	-

Klebsiella pneumoniae	W-KP55	-	-
Klebsiella pneumoniae	W-KP56	-	-
Klebsiella pneumoniae	W-KP57	-	-
Klebsiella pneumoniae	W-KP58	-	-
Klebsiella pneumoniae	W-KP59	-	-
Klebsiella pneumoniae	W-KP80	-	-
Klebsiella pneumoniae	W-KP81	-	-
Klebsiella pneumoniae	W-KP84	-	-
Klebsiella pneumoniae	W-KP85	-	-
Klebsiella pneumoniae	W-KP87	-	-
Klebsiella pneumoniae	W-KP88	-	-
Klebsiella pneumoniae	W-KP89	-	-
Klebsiella pneumoniae	W-KP91	-	-
Klebsiella pneumoniae	W-KP92	-	-
Klebsiella pneumoniae	W-KP94	-	-
Klebsiella pneumoniae	W-KP95	-	-
Klebsiella pneumoniae	W-KP96	-	-
Klebsiella pneumoniae	W-KP97	-	-
Klebsiella pneumoniae	W-KP98	-	-

Supplementary Table S3. General features of putative ORFs of phage P-KP2 with the best matches in non-redundant protein sequence database from NCBI.

ORF		bp		Length (aa)	Protein size (kDa)	pI	Annotated function	Representative similarity to proteins in database	E value	Query cover	Identity (positives)	Accession no.
		Start	Stop									
1+	AUG	164	583	139	16.120	8.49	single stranded DNA-binding protein	[*Klebsiella* phage KP15]	2e-96	100%	100% (100%)	YP_003580063.1
2+	AUG	583	759	58	6.320	4.24	hypothetical protein CPT_Matisse225	[*Klebsiella* phage Matisse]	7e-34	100%	100% (100%)	YP_009194469.1
3+	AUG	964	1116	50	5.861	10.29	hypothetical protein Lw1_gp222	[*Escherichia* phage Lw1]	1e-04	48%	83% (48%)	YP_008060743.1
4-	AUG	1195	1428	77	8.919	4.65	hypothetical protein KP15_188A	[*Klebsiella* phage KP15]	2e-47	100%	100% (100%)	YP_003934978.1
5-	GUG	1437	2936	499	13.891	9.21	Helicase	[*Klebsiella* phage KP27]	0.0	100%	99% (100%)	YP_007348859.1
6+	AUG	3007	3567	186	2.139	4.45	minor capsid protein inhibitor of protease	[*Klebsiella* phage KP27]	4e-134	100%	96% (100%)	YP_007348860.1
7+	AUG	3660	3971	103	12.131	5.31	hypothetical protein KP15_193	[*Klebsiella* phage KP15]	2e-71	100%	100% (100%)	YP_003580069.1
8+	AUG	4011	4580	189	21.540	6.21	hypothetical protein KP15_194	[*Klebsiella* phage KP15]	3e-138	100%	100% (100%)	YP_003580070.1
9+	AUG	4609	5670	353	37.692	4.06	phage tail fibers	[*Enterobacter* phage phiEap-3]	0.0	100%	98% (100%)	ALA45334.1
10+	AUG	5681	5956	91	10.772	4.24	hoc large outer capsid protein	[*Klebsiella* phage KP15]	3e-60	100%	100% (100%)	YP_003580072.1
11+	AUG	5970	6488	172	19.866	5.22	hypothetical protein	[*Escherichia* phage phT4A]	1e-124	100%	99% (100%)	ANN86488.1
12-	AUG	6521	6985	154	17.417	8.52	hypothetical protein KP15_199	[*Klebsiella* phage KP15]	7e-108	100%	100% (100%)	YP_003580075.1
13-	AUG	7035	7874	279	30.713	9.48	hypothetical protein KP15_200	[*Klebsiella* phage KP15]	0.0	100%	99% (100%)	YP_003580076.1
14+	AUG	7989	8864	291	33.928	7.89	hypothetical protein KP15_201	[*Klebsiella* phage KP15]	0.0	100%	97% (100%)	YP_003580077.1
15+	AUG	8972	9214	80	8.793	5.77	hypothetical protein ADS69_00235	[*Enterobacter* phage phiEap-3]	2e-52	100%	100% (100%)	ALA45340.1
16+	AUG	9211	9387	58	6.473	4.13	hypothetical protein KP15_203	[*Klebsiella* phage KP15]	8e-33	100%	100% (100%)	YP_003580079.1

17 +	AUG	9384	9710	108	12.270	5.55	hypothetical protein KP15_204	[*Klebsiella* phage KP15]	3e-72	100%	100% (100%)	YP_003580080.1Y
18 +	AUG	9712	10068	118	13.951	10.29	hypothetical protein KP15_205	[*Klebsiella* phage KP15]	4e-80	100%	100% (100%)	YP_003580081.1
19 +	AUG	10068	10592	174	19.792	9.69	Srd postulated decoy of host sigma70	[*Klebsiella* phage KP15]	7e-126	100%	100% (100%)	YP_003580082.1
20 +	AUG	10596	11108	170	19.222	9.02	RNA ligase 2	[*Enterobacter* phage phiEap-3]	3e-105	87%	87% (100%)	ALA45345.1
21 +	GUG	11105	11614	169	18.978	7.19	RNA ligase 2	[*Enterobacter* phage phiEap-3]	4e-119	100%	100% (100%)	ALA45345.1
22 +	AUG	11604	12185	193	21.658	6.97	hypothetical protein KP15_208	[*Klebsiella* phage KP15]	6e-139	100%	99% (100%)	YP_003580084.1
23 +	AUG	12179	12697	172	20.068	6.23	hypothetical protein KP15_209	[*Klebsiella* phage KP15]	5e-123	100%	98% (100%)	YP_003580085.1
24 +	AUG	12775	13035	86	9.652	4.07	hypothetical protein KP27_263	[*Klebsiella* phage KP27]	4e-55	100%	100% (100%)	YP_007348878.1
25 +	AUG	13045	13494	149	17.074	8.51	hypothetical protein KP27_264	[*Klebsiella* phage KP27]	1e-105	100%	100% (100%)	YP_007348879.1
26 +	AUG	13522	14955	477	53.326	6.83	nicotinamide phosphoribosyl transferase	[*Klebsiella* phage Miro]	0.0	100%	100% (100%)	YP_003580088.1
27 +	GUG	14942	15244	100	11.435	9.02	hypothetical protein KP27_266	[*Klebsiella* phage KP27]	1e-65	100%	99% (100%)	YP_007348881.1
28 +	AUG	15241	16092	283	33.355	6.15	DNA adenine methylase	[*Escherichia* phage phT4A]	0.0	100%	100% (100%)	ANN86504.1
29 +	AUG	16101	16376	91	10.394	7.19	NrdC thioredoxin	[*Klebsiella* phage KP15]	2e-61	100%	100% (100%)	YP_003580091.1
30 +	AUG	16408	16890	160	18.526	9.18	recombination endonuclease VII	[*Klebsiella* phage KP15]	6e-117	100%	100% (100%)	YP_003580092.1
31 +	AUG	16887	19010	707	79.942	6.75	ribonucleotide reductase of class III (anaerobic), large subunit	[*Klebsiella* phage PMBT1]	0.0	100%	99% (100%)	SCO64826.1
32 +	AUG	19078	19320	80	8.773	9.47	hypothetical protein ADS69_00251	[*Enterobacter* phage phiEap-3]	2e-51	100%	100% (100%)	ALA45356.1
33 +	AUG	19320	19814	164	19.103	9.41	hypothetical protein CPT_Matisse256	[*Klebsiella* phage Matisse]	8e-117	100%	100% (100%)	YP_009194500.1
34 +	AUG	19814	20020	68	8.189	10.11	hypothetical protein KP27_273	[*Klebsiella* phage KP27]	4e-42	100%	100% (100%)	YP_007348888.1
35 +	AUG	20041	20292	83	9.609	4.77	hypothetical protein KP27_274	[*Klebsiella* phage KP27]	5e-56	100%	100% (100%)	YP_007348889.1
36	AUG	20353	20547	64	7.663	8.82	hypothetical protein	[*Klebsiella* phage KP15]	2e-38	100%	100% (100%)	YP_003580100.1

+							KP15_224					
37 +	AUG	20544	21071	175	19.730	5.59	hypothetical protein KP15_225	[*Klebsiella* phage KP15]	8e-122	100%	99% (100%)	YP_003580101.1
38 +	AUG	21068	21559	163	18.681	8.82	anaerobic nucleotide reductase subunit	[*Enterobacter* phage phiEap-3]	8e-117	100%	99% (100%)	ALA45363.1
39 +	AUG	21629	22588	319	35.290	9.23	hypothetical protein KP27_278	[*Klebsiella* phage KP27]	0.0	100%	98% (100%)	YP_007348893.1
40 +	AUG	22631	23299	222	25.698	4.65	hypothetical protein KP15_228	[*Klebsiella* phage KP15]	1e-157	100%	99% (100%)	YP_003580104.1
41 +	AUG	23259	23540	93	10.848	6.37	glutaredoxin	[*Klebsiella* phage KP27]	3e-62	100%	100% (100%)	YP_007348895.1
42 +	AUG	23537	23836	99	11.794	9.42	hypothetical protein KP15_230	[*Klebsiella* phage KP15]	1e-66	100%	100% (100%)	YP_003580106.1
43 +	AUG	23916	27689	125 7	135.246	6.65	long tail fiber proximal subunit	[*Klebsiella* phage Matisse]	0.0	100%	99% (100%)	YP_009194510.1
44 +	AUG	27689	28813	374	41.046	4.55	hinge connector of long tail fiber proximal connector	[*Klebsiella* phage KP27]	0.0	100%	99% (100%)	YP_007348898.1
45 +	AUG	28863	29531	222	23.291	8.89	hinge connector of long tail fiber distal connector	[*Enterobacter* phage phiEap-3]	3e-159	100%	100% (100%)	ALA45370.1
46 +	AUG	29540	33259	123 9	130.386	7.04	tail fiber protein	[*Escherichia* phage phT4A]	0.0	97%	82% (97%)	ANN86512.1
47 +	AUG	33301	33702	133	15.816	4.99	hypothetical protein JD18_241	[*Klebsiella* phage JD18]	5e-83	100%	86%(100%)	YP_009190822.1
48 +	AUG	33782	34429	215	24.768	8.64	T holin lysis mediator	[*Klebsiella* phage KP27]	9e-159	100%	99% (100%)	YP_007348902.1
49	UUG	34430	34588	52	6.057	9.63	hypothetical protein KP15_236A	[*Klebsiella* phage KP15]	1e-30	100%	100% (100%)	YP_003934979.1
50 -	AUG	34696	34986	96	11.406	4.72	hypothetical protein	[*Escherichia* phage phT4A]	4e-66	100%	100% (100%)	ANN86518.1
51 -	AUG	34986	35783	265	30.518	6.02	hypothetical protein KP27_291	[*Klebsiella* phage KP27]	0.0	100%	100% (100%)	YP_007348905.1
52 -	AUG	35864	36091	75	8.886	9.14	hypothetical protein KP27_292	[*Klebsiella* phage KP27]	7e-50	100%	100% (100%)	YP_007348906.1
53 -	AUG	36088	36420	110	12.542	4.52	hypothetical protein CPT_Miro273	[*Klebsiella* phage Miro]	8e-76	100%	100% (100%)	AKU44857.1
54 -	AUG	36395	36529	44	5.336	10.44	hypothetical protein CPT_Miro274	[*Klebsiella* phage Miro]	1e-22	100%	100% (100%)	AKU44858.1
55 -	AUG	36574	36822	82	9.200	6.12	hypothetical protein KP15_238A	[*Klebsiella* phage KP15]	8e-50	100%	100% (100%)	YP_003934981.1
56 -	AUG	36859	37296	145	17.003	9.74	nucleoid disruption protein	[*Klebsiella* phage KP15]	9e-104	100%	100% (100%)	YP_003580115.1

57<br>-	AUG	37271	37645	124	14.110	9.77	hypothetical protein KP15_240	[*Klebsiella* phage KP15]	3e-83	100%	98% (100%)	YP_003580116.1
58<br>-	AUG	37691	38590	299	34.030	9.26	rllB protein	[*Klebsiella* phage KP27]	0.0	100%	99% (100%)	YP_007348913.1
59<br>-	AUG	38590	40851	753	86.966	8.54	phage rIIA lysis inhibitor	[*Klebsiella* phage PMBT1]	0.0	100%	99% (100%)	SCO64579.1
60<br>-	AUG	40862	41098	78	9.133	8.88	hypothetical protein CPT_Miro3	[*Klebsiella* phage Miro]	8e-48	100%	95% (100%)	AKU44587.1
61<br>-	AUG	41102	41656	184	21.284	9.62	hypothetical protein KP27_003	[*Klebsiella* phage KP27]	9e-135	100%	100% (100%)	YP_007348640.1
62<br>-	AUG	41728	43014	428	48.883	6,92	DNA topoisomerase medium subunit	[*Klebsiella* phage KP27]	0.0	100%	100% (100%)	YP_007348641.1
63<br>-	AUG	43014	44912	632	70.988	6.58	DNA topoisomerase large subunit	[*Klebsiella* phage KP27]	0.0	100%	100% (100%)	YP_007348642.1
64<br>-	AUG	44980	45144	54	6.226	8.16	hypothetical protein GAP161_006	[*Cronobacter* phage vB_CsaM_GAP161]	1e-30	100%	96% (100%)	YP_006986282.1
65<br>-	AUG	45144	45584	146	17.604	4.77	transcriptional regulator	[*Escherichia* phage phT4A]	3e-106	100%	100% (100%)	ANN86274.1
66<br>-	AUG	45607	46191	194	22.056	6.24	hypothetical protein KP27_010	[*Klebsiella* phage KP27]	1e-141	100%	99% (100%)	YP_007348645.1
67<br>-	AUG	46178	46456	92	10.406	7.31	hypothetical protein	[*Escherichia* phage phT4A]	2e-63	100%	100% (100%)	ANN86276.1
68<br>-	AUG	46453	47049	198	22.497	4.99	hypothetical protein CPT_Matisse11	[*Klebsiella* phage Matisse]	6e-147	100%	100% (100%)	YP_009194255.1
69<br>-	AUG	47089	47418	109	12.543	9.34	hypothetical protein KP27_014	[*Klebsiella* phage KP27]	3e-75	100%	100% (100%)	YP_007348648.1
70<br>-	AUG	47478	47702	74	8.723	6.36	hypothetical protein KP27_016	[*Klebsiella* phage KP27]	2e-46	100%	100% (100%)	YP_007348649.1
71<br>-	AUG	47776	48039	87	10.071	9.65	hypothetical protein KP15_13B	[*Klebsiella* phage KP15]	1e-56	100%	100% (100%)	YP_003934935.1
72<br>-	AUG	48128	48427	99	11.559	8.12	hypothetical protein KP15_14	[*Klebsiella* phage KP15]	1e-65	100%	100% (100%)	YP_003579890.1
73<br>-	AUG	48427	48651	74	8.372	9.65	hypothetical protein KP27_022	[*Klebsiella* phage KP27]	1e-47	100%	100% (100%)	YP_007348654.1
74<br>-	AUG	48655	48804	49	5.612	8.22	hypothetical protein KP15_14B	[*Klebsiella* phage KP15]	6e-26	100%	100% (100%)	YP_003934937.1
75<br>-	AUG	48871	49152	93	10.587	8.42	hypothetical protein KP15_15	[*Klebsiella* phage KP15]	2e-60	100%	99% (100%)	YP_003579891.1
76<br>-	AUG	49149	49820	223	26.005	5.17	DexA exonuclease A	[*Klebsiella* phage KP15]	5e-165	100%	100% (100%)	YP_003579892.1
77<br>-	AUG	49817	50101	94	10.867	4.85	hypothetical protein KP27_026	[*Klebsiella* phage KP27]	1e-63	100%	100% (100%)	YP_007348658.1

78 -	AUG	50102	50410	102	11.790	5.09	hypothetical protein KP15_18	[*Klebsiella* phage KP15]	1e-67	100%	100% (100%)	YP_003579894.1
79 -	AUG	50435	51763	442	50.617	7.04	dda DNA helicase	[*Klebsiella* phage KP15]	0.0	100%	100% (100%)	YP_003579895.1
80 -	AUG	51760	52050	96	11.327	9.11	hypothetical protein KP15_20	[*Klebsiella* phage KP15]	3e-63	100%	100% (100%)	YP_003579896.1
81 -	AUG	52232	52768	178	20.634	4.72	dCTP pyrophosphatase	[*Klebsiella* phage KP15]	9e-131	100%	100% (100%)	YP_003579897.1+
82 -	AUG	52776	53801	341	39.607	8.63	DNA primase subunit	[*Klebsiella* phage KP27]	0.0	100%	100% (100%)	YP_007348664.1
83 -	AUG	53805	54065	86	9.562	6.32	hypothetical protein KP27_032	[*Klebsiella* phage KP27]	2e-57	100%	100% (100%)	YP_007348665.1
84 -	AUG	54121	54618	165	18.879	9.65	hypothetical protein ADS69_00027	[*Enterobacter* phage phiEap-3]	3e-116	100%	98% (100%)	ALA45132.1
85 -	AUG	54624	55700	358	40.595	4.53	methyltransferase	[*Escherichia* phage Lw1]	2e-155	67%	87% (92%)	YP_008060555.1
86 -	AUG	55726	55962	78	8.527	9.56	hypothetical protein KP15_26A	[*Klebsiella* phage KP15]	7e-46	100%	100% (100%)	YP_003934939.1
87 -	AUG	56054	56308	84	9.516	4.27	hypothetical protein KP15_27	[*Klebsiella* phage KP15]	4e-53	100%	100% (100%)	YP_003579903.1
88 -	AUG	56388	56657	89	10.068	9.83	hypothetical protein KP15_27A	[*Klebsiella* phage KP15]	5e-55	100%	100% (100%)	YP_003934940.1
89 -	AUG	56701	58137	478	53,617	5.83	replication and recombination DNA helicase	[*Klebsiella* phage KP27]	0.0	100%	100% (100%)	YP_007348673.1+
90 -	AUG	58147	58467	106	12.368	5.28	membrane-associat ed initiation of head vertex	[*Klebsiella* phage KP15]	2e-70	100%	100% (100%)	YP_003579905.1
91 -	AUG	58505	59662	385	43.093	4.79	RecA-like recombination protein	[*Klebsiella* phage KP27]	0.0	100%	100% (100%)	YP_007348675.1
92 -	AUG	59737	62436	899	104.337	5.96	DNA polymerase	[*Klebsiella* phage KP27]	0.0	100%	99% (100%)	YP_007348676.1
93 -	AUG	62483	62812	109	12.637	8.17	hypothetical protein CPT_Miro40	[*Klebsiella* phage Miro]	6e-73	100%	100% (100%)	AKU44624.1
94 -	AUG	62809	63096	95	11.219	5.22	hypothetical protein CPT_Matisse40	[*Klebsiella* phage Matisse]	7e-63	100%	99% (100%)	YP_009194284.1
95 -	AUG	63165	63527	120	14.344	9.52	RegA	[*Klebsiella* phage KP27]	9e-83	100%	100% (100%)	YP_007348679.1

96 -	AUG	63531	64094	187	22.054	5.21	clamp-loader subunit	[*Klebsiella* phage KP15]	6e-133	100%	100% (100%)	YP_003579912.1
97 -	AUG	64097	65095	332	37.166	6.26	clamp-loader subunit	[*Klebsiella* phage KP15]	0.0	100%	100% (100%)	YP_003579913.1
98 -	AUG	65154	65819	221	24.497	4.76	sliding clamp DNA polymerase	[*Klebsiella* phage KP15]	6e-156	100%	100% (100%)	YP_003579915.1
99 -	AUG	65846	66115	89	10.605	8.39	RpbA RNA polymerase binding protein	[*Klebsiella* phage KP15]	2e-57	100%	100% (100%)	YP_003579916.1
100 -	AUG	66171	67142	323	35.327	4.56	ssDNA binding protein	[*Enterobacter* phage phiEap-3]	0.0	100%	100% (100%)	ALA45149.1
101 -	GUG	67158	67769	203	24.307	9.26	helicase assembly protein	[*Klebsiella* phage Matisse]	4e-142	100%	100% (100%)	YP_009194291.1
102 -	AUG	67814	68059	81	8.942	4.32	late promoter transcription	[*Klebsiella* phage KP15]	2e-52	100%	100% (100%)	YP_003579919.1
103 -	AUG	68063	68326	87	10.006	5.07	DsbA dsDNA binding	[*Klebsiella* phage KP15]	1e-55	100%	100% (100%)	YP_003579920.1
104 -	AUG	68336	69271	311	36.619	5.76	RnaseH	[*Klebsiella* phage KP15]	0.0	100%	100% (100%)	YP_003579921.1
105 +	AUG	69585	70103	172	20.039	4.77	RNA polymerase sigma factor for late transcription	[*Escherichia* phage phT4A]	6e-126	100%	100% (100%)	ANN86315.1
106 +	AUG	70100	70315	71	82.07	3.63	hypothetical protein KP15_46A	[*Klebsiella* phage KP15]	1e-41	100%	100% (100%)	YP_003934941.1
107 +	AUG	70308	70634	108	86012.	9.22	hypothetical protein KP15_47	[*Klebsiella* phage KP15]	2e-72	100%	100% (100%)	YP_003579923.1
108 +	AUG	70686	71717	268	31.050	5.25	recombination-relat ed endonuclease	[*Escherichia* phage phT4A]	0.0	100%	100% (100%)	ANN86318.1
109 +	AUG	71704	71991	95	11.142	3.95	hypothetical protein KP15_49	[*Klebsiella* phage KP15]	2e-62	100%	100% (100%)	YP_003579925.1
110 +	AUG	71975	73672	565	64.092	7.94	recombination endonuclease subunit	[*Klebsiella* phage KP27]	0.0	100%	100% (100%)	YP_007348694.1
111 +	AUG	73672	73863	63	7.748	4.86	hypothetical protein KP15_50A	[*Klebsiella* phage KP15]	3e-35	100%	97% (100%)	YP_003934942.1
112 +	AUG	73937	75646	569	63.575	9.56	hypothetical protein KP15_51	[*Klebsiella* phage KP15]	0.0	100%	99% (100%)	YP_003579927.1
113 +	AUG	75706	75996	96	11.419	8.13	hypothetical protein KP27_068	[*Klebsiella* phage KP27]	5e-65	100%	100% (100%)	YP_007348697.1

114 +	AUG	75993	76223	76	8.592	3.71	hypothetical protein KP15_52A	[*Klebsiella* phage KP15]	8e-48	100%	100% (100%)	YP_003934943.1
115 +	AUG	76220	76477	85	10.031	7.04	hypothetical protein KP15_52B	[*Klebsiella* phage KP15]	9e-56	100%	100% (100%)	YP_003934944.1
116 +	AUG	76474	77052	192	21.234	6.49	dihydrofolate reductase	[*Escherichia* phage phT4A]	4e-140	100%	100% (100%)	ANN86327.1
117 +	AUG	77054	77929	291	33.017	5.09	thymidylate synthetase	[*Klebsiella* phage KP15]	0.0	100%	100% (100%)	YP_003579930.1
118 +	AUG	77969	80221	750	85.085	5.89	NrdA ribonucleotide reductase A subunit	[*Klebsiella* phage KP15]	0.0	100%	99% (100%)	YP_003579931.1
119 +	AUG	80258	81433	391	45.508	5.64	aerobic ribonucleotide reductase B subunit	[*Klebsiella* phage KP27]	0.0	100%	100% (100%)	YP_007348703.1
120 +	AUG	81405	81725	106	11.964	8.08	hypothetical protein KP15_58	[*Klebsiella* phage KP15]	2e-74	100%	100% (100%)	YP_003579934.1
121 +	AUG	81729	82094	121	14.673	9.21	DenA endonuclease II	[*Klebsiella* phage KP15]	3e-85	100%	100% (100%)	YP_003579935.1
122 +	AUG	82075	83232	385	44.198	4.97	RNA ligase 1	[*Klebsiella* phage KP27]	0.0	100%	100% (100%)	YP_007348706.1
123 +	UUG	83289	83552	87	10.053	8.82	i-spanin	[*Klebsiella* phage Matisse]	4e-54	100%	100% (100%)	YP_009194314.1
124 +	GUG	83654	83866	70	8.171	8.81	o-spanin	[*Klebsiella* phage Matisse]	3e-44	100%	100% (100%)	YP_009194315.1
125 +	AUG	83851	84024	57	7.056	5.25	hypothetical protein KP27_081	[*Klebsiella* phage KP27]	3e-31	100%	98% (100%)	YP_007348709.1
126 +	AUG	84021	84905	294	33.751	6.56	3'phosphatase, 5'polynucleotide kinase	[*Klebsiella* phage KP27]	0.0	100%	100% (100%)	YP_007348710.1
127 +	AUG	84958	85245	95	10.653	6.34	hypothetical protein KP27_083	[*Klebsiella* phage KP27]	9e-65	100%	100% (100%)	YP_007348711.1
128 +	AUG	85242	85493	83	9.379	4.46	hypothetical protein CPT_Miro76	[*Klebsiella* phage Miro]	6e-52	100%	100% (100%)	AKU44660.1
129 +	AUG	85486	85770	94	10.745	6.04	hypothetical protein CPT_Matisse76	[*Klebsiella* phage Matisse]	6e-64	100%	100% (100%)	YP_009194320.1
130 +	AUG	85760	86266	168	19.207	7.37	cytidine deaminase	[*Klebsiella* phage Matisse]	7e-125	100%	100% (100%)	YP_009194321.1
131 +	AUG	86282	86623	113	12.318	6.13	head assembly chaperone with GroEL	[*Klebsiella* phage Miro]	4e-76	100%	100% (100%)	AKU44663.1

132 +	AUG	86634	86885	83	9.420	8.12	hypothetical protein KP15_69A	[*Klebsiella* phage KP15]	2e-53	100%	100% (100%)	YP_003934946.1
133 +	AUG	87200	87481	93	11.106	4.35	hypothetical protein KP15_70	[*Klebsiella* phage KP15]	2e-63	100%	100% (100%)	YP_003579946.1
134 +	AUG	87491	87913	140	16.399	8.87	hypothetical protein ADS69_00078	[*Enterobacter* phage phiEap-3]	2e-95	100%	99% (100%)	ALA45183.1
135 +	AUG	88096	88236	46	5.637	10.05	hypothetical protein ADS69_00079	[*Enterobacter* phage phiEap-3]	7e-21	89%	98% (89%)	ALA45184.1
136 +	GUG	88196	88345	49	5.148	3.95	hypothetical protein KP15_76A	[*Klebsiella* phage KP15]	4e-21	95%	94% 95%)	YP_003934951.1
137 +	AUG	88347	88568	73	8.728	4.16	hypothetical protein KP27_103	[*Klebsiella* phage KP27]	2e-46	100%	100% (100%)	YP_007348729.1
138 +	UUG	88565	89179	204	23.307	5.40	hypothetical protein KP27_104	[*Klebsiella* phage KP27]	5e-151	100%	99% (100%)	YP_007348730.1
139 +	AUG	89160	90689	509	58.200	4.99	DNA ligase	[*Klebsiella* phage KP15]	0.0	100%	100% (100%)	YP_003579955.1
140 +	AUG	90766	91335	189	21.690	6.92	hypothetical protein KP15_81	[*Klebsiella* phage KP15]	2e-139	100%	100% (100%)	YP_003579957.1
141 +	AUG	91424	91627	67	7.770	8.90	hypothetical protein CPT_Matisse95	[*Klebsiella* phage Matisse]	5e-43	100%	100% (100%)	YP_009194339.1
142 +	AUG	91630	91806	58	6.605	5.19	hypothetical protein CPT_Matisse96	[*Klebsiella* phage Matisse]	3e-34	100%	100% (100%)	YP_009194340.1
143 +	AUG	91803	91967	54	6.307	5.33	hypothetical protein CPT_Matisse97	[*Klebsiella* phage Matisse]	2e-30	100%	100% (100%)	YP_009194341.1
144 +	AUG	92048	92338	96	10.823	4.11	hypothetical protein CPT_Matisse98	[*Klebsiella* phage Matisse]	8e-64	100%	100% (100%)	YP_009194342.1
145 +	AUG	92349	92660	103	11.836	6.55	hypothetical protein CPT_Matisse99	[*Klebsiella* phage Matisse]	9e-69	100%	100% (100%)	YP_009194343.1
146 +	AUG	92647	93024	125	14.680	5.00	hypothetical protein KP15_85	[*Klebsiella* phage KP15]	3e-87	100%	100% (100%)	YP_003579961.1
147 +	AUG	93021	94130	369	42.012	4.93	baseplate hub subunit	[*Klebsiella* phage KP27]	0.0	100%	100% (100%)	YP_007348737.1
148 +	AUG	94200	94775	191	22.173	6.11	baseplate distal hub subunit	[*Klebsiella* phage KP15]	3e-137	100%	100% (100%)	YP_003579963.1
149 +	AUG	94778	96565	595	67.110	5.26	baseplate hub	[*Enterobacter* phage phiEap-3]	0.0	100%	99% (100%)	ALA45208.1
150 +	AUG	96576	97649	357	39.074	5.91	tail-tube assembly protein	[*Klebsiella* phage Matisse]	0.0	100%	100% (100%)	YP_009194348.1
151 +	AUG	97659	98522	287	32.042	4.68	baseplate subunit	[*Klebsiella* phage KP15]	0.0	100%	100% (100%)	YP_003579966.1

152 +	AUG	98551	99294	247	28.527	4.88	baseplate hub assembly protein	[*Klebsiella* phage KP15]	9e-180	100%	100% (100%)	YP_003579967.1
153 +	AUG	99291	99845	184	21.474	4.95	baseplate hub subunit	[*Klebsiella* phage KP15]	5e-135	100%	100% (100%)	YP_003579968.1
154 +	AUG	99846	100238	130	14.453	4.19	tail lysozyme	[*Klebsiella* phage KP15]	1e-89	100%	100% (100%)	YP_003579970.1
155 -	AUG	100273	100728	151	17.601	5.04	hypothetical protein KP15_95	[*Klebsiella* phage KP15]	3e-106	100%	97% (100%)	YP_003579971.1
156 -	AUG	100736	100960	74	8.351	5.18	hypothetical protein KP15_95A	[*Klebsiella* phage KP15]	2e-46	100%	97% (100%)	YP_003934954.1
157 -	AUG	100962	101246	94	10.879	5.16	hypothetical protein KP15_96	[*Klebsiella* phage KP15]	9e-61	100%	100% (100%)	YP_003579972.1
158 -	AUG	101318	101602	94	10.811	4.23	hypothetical protein KP15_96A	[*Klebsiella* phage KP15]	6e-62	100%	100% (100%)	YP_003934955.1
159 -	AUG	101618	102088	156	17.696	4.18	hypothetical protein CPT_Matisse113	[*Klebsiella* phage Matisse]	4e-109	100%	100% (100%)	YP_009194357.1
160 -	AUG	102085	102243	52	5.859	8.23	hypothetical protein KP15_97A	[*Klebsiella* phage KP15]	6e-28	100%	100% (100%)	YP_003934956.1
161 -	GUG	102240	102890	216	24.736	4.68	hypothetical protein KP27_126	[*Klebsiella* phage KP27]	2e-156	100%	99% (100%)	YP_007348751.1
162 -	AUG	102929	105145	738	82.377	5.13	hypothetical protein KP15_100	[*Klebsiella* phage KP15]	0.0	100%	100% (100%)	YP_003579976.1
163 -	AUG	105153	105473	106	12.262	4.49	hypothetical protein KP15_101	[*Klebsiella* phage KP15]	2e-71	100%	100% (100%)	YP_003579977.1
164 -	AUG	105473	105988	171	20.099	4.57	hypothetical protein CPT_Matisse118	[*Klebsiella* phage Matisse]	3e-127	100%	100% (100%)	YP_009194362.1
165 -	AUG	105985	106227	80	9.06	5.50	hypothetical protein CPT_Matisse119	[*Klebsiella* phage Matisse]	8e-53	100%	100% (100%)	YP_009194363.1
166 -	AUG	106214	106501	95	11.058	4.31	hypothetical protein CPT_Matisse120	[*Klebsiella* phage Matisse]	4e-64	100%	100% (100%)	YP_009194364.1
167 -	AUG	106708	106986	92	10.603	5.82	hypothetical protein GAP161_115	[*Cronobacter* phage vB_CsaM_GAP161]	7e-63	100%	100% (100%)	YP_006986393.1
168 -	AUG	107045	107956	303	34.182	8.31	DNA methyltransferase	[*Klebsiella* phage Matisse]	0.0	100%	100% (100%)	YP_009194367.1
169 -	AUG	107953	108210	85	9.503	4.19	hypothetical protein KP27_138	[*Klebsiella* phage KP27]	4e-54	100%	100% (100%)	YP_007348761.1
170 -	AUG	108210	108455	81	8.984	5.73	hypothetical protein CPT_Matisse125	[*Klebsiella* phage Matisse]	1e-53	100%	100% (100%)	YP_009194369.1
171 -	AUG	108452	109459	335	38.083	7.30	hypothetical protein KP15_106	[*Klebsiella* phage KP15]	0.0	100%	99% (100%)	YP_003579982.1

172 -	AUG	109459	109836	125	14.555	8.55	hypothetical protein CPT_Matisse127	[*Klebsiella* phage Matisse]	1e-87	100%	99% (100%)	YP_009194371.1
173 -	AUG	109833	110060	75	8.756	8.30	hypothetical protein CPT_Miro128	[*Klebsiella* phage Miro]	7e-48	100%	99% (100%)	AKU44712.1
174 -	AUG	110057	110662	201	22.284	7.96	hypothetical protein KP15_109	[*Klebsiella* phage KP15]	1e-142	100%	99% (100%)	YP_003579985.1
175 -	AUG	110665	110937	90	10.476	5.29	hypothetical protein CPT_Matisse130	[*Klebsiella* phage Matisse]	8e-61	100%	99% (100%)	YP_009194374.1
176 -	AUG	110934	111128	64	7.036	5.09	hypothetical protein RB16p128	[*Escherichia* virus RB16]	7e-38	100%	100% (100%)	YP_003858428.1
177 _	AUG	111303	112364	353	40.851	6.99	ADP-ribose pyrophosphatase	[*Klebsiella* phage PMBT1]	0.0	100%	99% (100%)	SCO64710.1
178 -	AUG	112541	113455	304	34.070	8.57	hypothetical protein KP15_111	[*Klebsiella* phage KP15]	0.0	100%	100% (100%)	YP_003579987.1
179 -	AUG	113527	113949	140	15.933	6.18	hypothetical protein KP27_148	[*Klebsiella* phage KP27]	2e-98	100%	100% (100%	YP_007348771.1
180 -	AUG	113921	114202	93	10.803	8.49	rI antiholin	[*Enterobacter* phage phiEap-3]	5e-65	100%	77.42% (100%)	YP_009194955.1
181 -	AUG	114309	114701	130	15.480	9.66	hypothetical protein KP27_150	[*Klebsiella* phage KP27]	6e-92	100%	100% (100%)	YP_007348773.1
182 -	AUG	114718	115326	202	22.964	5.90	thymidine kinase	[*Klebsiella* phage KP27]	9e-150	100%	100% (100%)	YP_007348774.1
183 -	AUG	115319	115606	95	10.722	9.16	hypothetical protein	[*Klebsiella* phage PMBT1]	2e-63	100%	100% (100%)	SCO64716.1
184 -	AUG	115609	116082	157	17.029	7.78	hypothetical protein KP27_155	[*Klebsiella* phage KP27]	2e-111	100%	99% (100%)	YP_007348778.1
185 -	AUG	116167	116313	48	5.588	9.79	hypothetical protein	[*Klebsiella* phage PMBT1]	1e-26	100%	100% (100%)	SCO64718.1
186 -	AUG	116264	116380	38	4.247	3.99	hypothetical protein CPT_Matisse143	[*Klebsiella* phage Matisse]	2e-19	100%	100% (100%)	YP_009194387.1
187 -	AUG	116380	116703	107	12.265	7.76	hypothetical protein KP15_117	[*Klebsiella* phage KP15]	3e-73	100%	100% (100%)	YP_003579993.1
188 -	AUG	116713	117303	196	22.726	4.75	hypothetical protein KP15_118	[*Klebsiella* phage KP15]	4e-145	100%	99% (100%)	YP_003579994.1
189 -	AUG	117300	117446	48	5.498	7.15	hypothetical protein KP15_118A	[*Klebsiella* phage KP15]	1e-27	100%	100% (100%)	YP_003934964.1
190 -	AUG	117457	117852	131	15.645	5.06	hypothetical protein CPT_Miro147	[*Klebsiella* phage Miro]	1e-92	100%	100% (100%)	AKU44731.1
191 -	AUG	117920	118561	213	23.945	9.53	hypothetical protein ADS69_00146 protein, T4 GC1559	[*Enterobacter* phage phiEap-3]	3e-155	100%	99% (100%)	ALA45251.1

192 -	AUG	118626	119180	184	21.431	5.81	hypothetical protein KP15_121	[*Klebsiella* phage KP15]	1e-132	100%	99% (100%)	YP_003579997.1
193 -	AUG	119177	119362	61	7.166	5.17	hypothetical protein	[*Klebsiella* phage PMBT1]	2e-35	100%	100% (100%)	SCO64725.1
194 -	AUG	119606	120952	448	49.754	5.00	PhoH domain protein	[*Klebsiella* phage Matisse]	0.0	100%	100% (100%)	YP_009194395.1
195 -	AUG	120962	121486	174	19.675	9.53	homing endonuclease	[*Klebsiella* phage Miro]	5e-127	100%	100% (100%)	AKU44736.1
196 -	AUG	121486	121944	152	17.616	7.48	hypothetical protein KP15_125	[*Klebsiella* phage KP15]	3e-111	100%	100% (100%)	YP_003580001.1
197 -	AUG	121976	122371	131	14.727	9.66	endolysin	[*Cronobacter* phage vB_CsaM_leB]	3e-86	100%	95% (98%)	AOG16285.1
198 -	AUG	122439	122840	133	15.731	6.15	hypothetical protein KP27_167	[*Klebsiella* phage KP27]	1e-92	100%	100% (100%)	YP_007348789.1
199 -	AUG	122895	123218	107	12.057	4.36	hypothetical protein CPT_Matisse156	[*Klebsiella* phage Matisse]	7e-73	100%	100% (100%)	YP_009194400.1
200 -	AUG	123258	123512	84	9.458	4.03	hypothetical protein KP27_169	[*Klebsiella* phage KP27]	2e-55	100%	100% (100%)	YP_007348791.1
201 -	AUG	123591	123851	86	9.357	6.99	hypothetical protein KP27_170	[*Klebsiella* phage KP27]	3e-56	100%	100% (100%)	YP_007348792.1
202 -	AUG	123853	124284	143	16.046	4.67	hypothetical protein KP27_171	[*Klebsiella* phage KP27]	2e-103	100%	100% (100%)	YP_007348793.1
203 -	AUG	124296	124550	84	9.705	8.55	hypothetical protein ADS69_00156	[*Enterobacter* phage phiEap-3]	1e-54	100%	98% (100%)	ALA45261.1
204 -	AUG	124564	124737	57	6.506	10.03	hypothetical protein CPT_Matisse160	[*Klebsiella* phage Matisse]	4e-33	100%	98% (100%)	YP_009194404.1
205 -	AUG	124734	125081	115	13.233	5.06	hypothetical protein KP27_173	[*Klebsiella* phage KP27]	2e-78	100%	100% (100%)	YP_007348795.1
206 -	AUG	125062	125409	115	13.462	5.95	hypothetical protein	[*Escherichia* phage phT4A]	1e-76	97%	99% (97%)	ANN86416.1
207 -	AUG	125406	126551	381	42.627	5.29	hypothetical protein ADS69_00161	[*Enterobacter* phage phiEap-3]	0.0	100%	99% (100%)	ALA45266.1
208 -	AUG	126562	126765	67	7.899	7.34	hypothetical protein CPT_Matisse165	[*Klebsiella* phage Matisse]	2e-42	100%	100% (100%)	YP_009194409.1
209 -	AUG	126765	127022	85	10.354	9.65	hypothetical protein KP15_136	[*Klebsiella* phage KP15]	1e-55	100%	100% (100%)	YP_003934967.1
210 -	AUG	127082	127252	56	6.956	8.16	hypothetical protein KP15_136A	[*Klebsiella* phage KP15]	8e-35	100%	100% (100%)	YP_003934968.1
211 -	AUG	127249	127440	63	7.377	3.91	hypothetical protein KP15_136B	[*Klebsiella* phage KP15]	2e-38	100%	100% (100%)	YP_003934969.1

212-	AUG	127443	127592	49	5.715	5.03	hypothetical protein ADS69_00167	[*Enterobacter* phage phiEap-3]	4e-28	100%	100% (100%)	ALA45272.1
213-	AUG	127589	127840	83	9.753	7.03	hypothetical protein	[*Klebsiella* phage PMBT1]	1e-51	100%	98% (100%)	SCO64745.1
214-	AUG	127912	128190	92	10.397	4.53	hypothetical protein CPT_Matisse171	[*Klebsiella* phage Matisse]	1e-60	100%	100% (100%)	YP_009194415.1
215-	AUG	128261	128593	110	12.716	7.09	hypothetical protein CPT_Matisse172	[*Klebsiella* phage Matisse]	7e-76	100%	99% (100%)	YP_009194416.1
216-	AUG	128929	129504	191	22.283	6.22	hypothetical protein CPT_Miro171	[*Klebsiella* phage Miro]	9e-141	100%	99% (100%)	AKU44755.1
217-	AUG	129512	129910	132	15.053	6.99	hypothetical protein	[*Escherichia* phage phT4A]	2e-94	100%	100% (100%)	ANN86426.1
218-	AUG	130085	130285	66	7.572	7.97	hypothetical protein KP27_187	[*Klebsiella* phage KP27]	9e-42	100%	100% (100%)	YP_007348809.1
219-	AUG	130362	131276	304	35.369	6.82	hypothetical protein	[*Klebsiella* phage PMBT1]	0.0	100%	99% (100%)	SCO64751.1
220-	AUG	131273	131800	175	20.559	6.36	hypothetical protein KP15_143	[*Klebsiella* phage KP15]	3e-129	100%	100% (100%)	YP_003580019.1
221-	AUG	131800	132123	107	12.053	6.11	hypothetical protein KP15_145	[*Klebsiella* phage KP15]	7e-74	100%	100% (100%)	YP_003580021.1
222-	AUG	132152	132409	84	9.585	5.15	hypothetical protein KP15_145A	[*Klebsiella* phage KP15]	3e-53	100%	99% (100%)	YP_003934973.1
223-	AUG	132411	132794	127	14.833	4.51	hypothetical protein KP27_193	[*Klebsiella* phage KP27]	5e-89	100%	100% (100%)	YP_007348814.1
224-	AUG	132805	133212	135	15.136	5.35	NudE nudix hydrolase	[*Klebsiella* phage KP15]	1e-96	100%	100% (100%)	YP_003580023.1
225-	AUG	133213	133458	81	9.382	5.05	hypothetical protein KP27_195	[*Klebsiella* phage KP27]	4e-52	100%	100% (100%)	YP_007348816.1
226-	AUG	133455	133706	83	9.307	5.17	hypothetical protein	[*Escherichia* phage phT4A]	2e-56	100%	100% (100%)	ANN86436.1
227-	AUG	133699	133992	97	11.214	3.94	hypothetical protein KP15_147A	[*Klebsiella* phage KP15]	4e-67	100%	100% (100%)	YP_003934974.1
228-	AUG	133989	134324	111	12.582	8.72	hypothetical protein CPT_Matisse187	[*Klebsiella* phage Matisse]	1e-76	100%	99% (100%)	YP_009194431.1
229-	AUG	134335	134463	42	4.614	9.29	hypothetical protein KP15_147B	[*Klebsiella* phage KP15]	8e-20	100%	100% (100%)	YP_003934975.1
230-	AUG	134565	134705	46	5.252	8.32	hypothetical protein ADS69_00187	[*Enterobacter* phage phiEap-3]	6e-27	100%	100% (100%)	ALA45292.1
231-	AUG	134771	135310	179	20.832	9.49	hypothetical protein KP27_201	[*Klebsiella* phage KP27]	8e-131	100%	100% (100%)	YP_007348822.1

232 -	AUG	135318	135941	207	23.614	5.14	hypothetical protein KP27_202	[*Klebsiella* phage KP27]	2e-151	100%	100% (100%)	YP_007348823.1
233 -	AUG	135993	136253	86	9.175	4.11	chaperone for tail fiber formation	[*Klebsiella* phage KP27]	2e-52	100%	99%(100%)	YP_007348824.1
234 -	AUG	136268	136963	231	26.845	7.23	deoxynucleotide monophosphate kinase	[*Klebsiella* phage PMBT1]	7e-170	100%	99%100%)	SCO64766.1
235 -	AUG	136963	137493	176	19.879	4.35	tail completion and sheath stabilizer protein	[*Klebsiella* phage KP15]	9e-129	100%	100% (100%)	YP_003580027.1
236 -	AUG	137863	138651	262	29.319	8.50	hypothetical protein KP27_206	[*Klebsiella* phage KP27]	0.0	100%	99%100%)	YP_007348827.1
237 -	AUG	138688	139542	284	32.858	10.13	DNA end protector protein	[*Klebsiella* phage KP27]	0.0	100%	100% (100%)	YP_007348828.1
238 -	AUG	139558	140025	155	18.733	9.72	head completion protein	[*Klebsiella* phage KP15]	3e-112	100%	100% (100%)	YP_003580031.1
239 +	GUG	140076	140630	184	21.723	4.71	baseplate wedge subunit	[*Klebsiella* phage KP27]	1e-133	100%	99%100%)	YP_007348830.1
240 +	AUG	140627	142396	589	64.314	4.97	baseplate hub subunit and tail lysozyme	[*Enterobacter* phage phiEap-3]	0.0	100%	100% (100%)	ALA45302.1
241 +	AUG	142401	144467	688	76.194	4.73	hypothetical protein CPT_Miro198	[*Klebsiella* phage Miro]	0.0	100%	99%100%)	AKU44782.1
242 +	AUG	144499	146424	641	71.177	4.35	phage baseplate wedge subunit (T4-like gp6)	[*Klebsiella* phage PMBT1]	0.0	100%	99%(100%)	SCO64774.1
243 +	AUG	146505	149591	1028	11.8675	4.92	baseplate wedge subunit	[*Escherichia* phage phT4A]	0.0	100%	99%(100%)	ANN86454.1
244 +	AUG	149592	150584	330	37.579	4.65	baseplate wedge subunit	[*Klebsiella* phage Matisse]	0.0	100%	100% (100%)	YP_009194447.1
245 +	AUG	150594	151457	287	31.038	4.96	baseplate wedge tail fiber connector	[*Escherichia* phage phT4A]	0.0	100%	99%(100%)	ANN86456.1
246 +	AUG	151454	153271	605	66.533	4.49	baseplate wedge subunit and tail pin	[*Enterobacter* phage phiEap-3]	0.0	100%	99%(100%)	ALA45308.1
247 +	AUG	153271	153939	222	24.392	4.69	baseplate wedge subunit tail pin	[*Klebsiella* phage KP27]	3e-162	100%	100% (100%)	YP_007348838.1
248 +	AUG	153949	155334	461	49.841	7.41	tail fibers protein	[*Escherichia* phage phT4A]	0.0	100%	99%(100%)	ANN86459.1
249 +	AUG	155348	157099	583	62.849	4.30	phage neck whiskers	[*Klebsiella* phage PMBT1]	0.0	100%	99%(100%)	SCO64781.1

250 +	AUG	157138	158064	308	33.596	4.62	neck protein	[*Klebsiella* phage KP15]	0.0	100%	100% (100%)	YP_003580045.1
251 +	AUG	158074	158820	248	28.934	4.10	neck protein	[*Klebsiella* phage KP15]	0.0	100%	100% (100%)	YP_003580046.1
252 +	AUG	158900	159724	274	31.916	5.76	tail sheath stabilizer and completion protein	[*Enterobacter* phage phiEap-3]	0.0	100%	99%(100%)	ALA45314.1
253 +	AUG	159724	160263	179	20.008	4.44	small terminase protein	[*Klebsiella* phage KP15]	3e-128	100%	100% (100%)	YP_003580048.1
254 +	AUG	160232	162061	609	69.656	5.69	large terminase protein	[*Klebsiella* phage KP15]	0.0	100%	100% (100%)	YP_003580049.1
255 +	AUG	162081	164072	663	71.994	4.80	phage tail sheath monomer	[*Klebsiella* phage PMBT1]	0.0	100%	100% (100%)	SCO64787.1
256 +	AUG	164121	164606	161	18.223	4.68	tail tube protein	[*Klebsiella* phage KP15]	8e-117	100%	100% (100%)	YP_003580053.1
257 +	AUG	164662	166236	524	60.723	5.15	portal vertex protein	[*Klebsiella* phage KP15]	0.0	100%	100% (100%)	YP_003580054.1
258 +	AUG	166236	166478	80	9.215	4.31	prohead core protein	[*Klebsiella* phage Matisse]	9e-46	100%	99%(100%)	YP_009194461.1
259 +	AUG	166487	166891	134	15.120	10.19	prohead core protein	[*Klebsiella* phage KP15]	6e-89	100%	100% (100%)	YP_003580056.1
260 +	AUG	166894	167541	215	23.505	5.03	prohead core and protease	[*Klebsiella* phage KP27]	1e-151	100%	99%(100%)	YP_007348851.1
261 +	AUG	167574	168362	262	29.278	4.33	prohead core protein	[*Klebsiella* phage KP15]	0.0	100%	100% (100%)	YP_003580058.1
262 +	AUG	168382	169950	522	56.121	5.05	major capsid protein	[*Klebsiella* phage KP15]	0.0	100%	100% (100%)	YP_003580059.1
263 +	AUG	170042	170680	212	24.131	9.78	homing endonuclease	[*Klebsiella* phage Matisse]	3e-152	100%	98% (100%)	YP_009194466.1
264 +	AUG	170713	172002	429	47.181	4.96	capsid vertex protein	[*Klebsiella* phage KP15]	0.0	100%	99% (100%)	YP_003580062.1

+, right orientation; -, left orientation.
